# Rapid sonication-assisted whole tissue clearing and immunostaining

**DOI:** 10.1038/s41598-025-18928-5

**Published:** 2025-10-08

**Authors:** Hoi Pan Harry Cheung, Marianne Lauwers, Zhengao Wang, Jianfeng Wang, Chengyun Ning, Dai Fei Elmer Ker, Dan Michelle Wang

**Affiliations:** 1 School of Biomedical Sciences, Faculty of Medicine, The Chinese University of Hong Kong, Sha Tin, New Territories, Hong Kong SAR China; 2 Institute for Tissue Engineering and Regenerative Medicine, The Chinese University of Hong Kong, Sha Tin, New Territories, Hong Kong SAR China; 3 Center for Neuromusculoskeletal Restorative Medicine, Hong Kong Science Park, Shatin, New Territories, Hong Kong SAR China; 4 Research Center of Biomass 3D Printing Materials, College of Materials and Energy, South China Agricultural University, Guangzhou, 510642 China; 5 College of Materials Science, Hunan University, Changsha, 410082 China; 6 School of Materials Science and Engineering, National Engineering Research Center for Tissue Restoration and Reconstruction, South China University of Technology, Guangzhou, 510641 China; 7 Department of Biomedical Engineering, Hong Kong Polytechnic University, Hung Hom, Kowloon, Hong Kong SAR China; 8 Department of Orthopaedics and Traumatology, Faculty of Medicine, The Chinese University of Hong Kong, Sha Tin, New Territories, Hong Kong SAR China

**Keywords:** Tissue clearing, Whole-tissue immunostaining, Optical imaging, Sonication, Biological techniques, Biophysical methods, Imaging

## Abstract

High-resolution mapping of three-dimensional structures in biological tissues is essential for understanding various biological processes. However, the optical heterogeneity of these tissues, marked by varying optical properties, causes light scattering and absorption, complicating imaging. Current tissue clearing methods can take between 48 h and 32 days, and the limited diffusion depth of fluorescent probes restricts whole-tissue imaging. This study introduces an innovative Sonication-Assisted Tissue Clearing and Immunofluorescent Staining method (SoniC/S), which combines low-frequency ultrasound with a commercial chemical clearing kit and iDISCO staining techniques. When tested on the soft tissue of mouse muscle, the dense collagenous tissue of rat tendon, and the heme-rich tissue of mouse spleen, SoniC/S achieved complete clearing in just 36 h and uniform labeling in 15 h. Overall, SoniC/S provides a rapid and effective approach for tissue clearing and deep immunostaining, facilitating high-resolution volumetric imaging in biological research.

**Supplementary Information:**

The online version contains supplementary material available at 10.1038/s41598-025-18928-5.

## Introduction

High-resolution mapping of three-dimensional (3D) structures within intact biological tissues is essential for advancing our understanding of various biological processes. This approach facilitates the investigation of structural alterations in tissues associated with diseases and provides valuable insights into disease progression and the underlying pathology^1–3^. Furthermore, it enables the precise localization of cells and their interactions within the tissue microenvironment, thereby elucidating communication pathways and signal transduction mechanisms involved in processes such as immune responses^4–6^.

Biological tissues are composed of heterogeneous substances with varying optical properties, which complicates the imaging process^7–13^. The heterogeneity in refractive indices (RIs) among these components leads to significant light scattering^8,14,15^. Additionally, light absorption by endogenous chromophores, such as heme, further attenuates light transmission^16^. Consequently, light scattering and absorption contribute to tissue opacity, presenting major challenges for volumetric imaging^17^. Tissue clearing techniques have emerged as methods to enhance the optical transparency of intact organs and organisms by minimizing light scattering and absorption^7,8,14,18^. The tissue clearing process typically encompasses several key steps. The initial step, tissue fixation, is essential for preserving structural and molecular integrity throughout the clearing procedure. The decolorization step removes endogenous pigments which absorb light^16^. The final step includes equilibrating the RI of the entire tissue. This will reduce light scattering and contributes to improved clearing efficacy and imaging quality as the light penetrates deeper into the samples^7,17,19^. Current tissue clearing methods can be classified into two categories: organic-solvent-based and aqueous-based techniques, depending on the chemical reagents employed. Both approaches aim to harmonize RIs and remove endogenous pigments within the tissue, thereby promoting deep light penetration suitable for high-resolution volumetric imaging^15,20^. Aqueous tissue clearing preserves morphological integrity using water-compatible solutions, while organic solvent methods displace lipids to achieve higher transparency^21^.

Tissue clearing techniques have significantly advanced high-resolution 3D imaging of intact biological tissue. However, several limitations remain such as prolonged processing times for tissue clearing and restricted diffusion depths of fluoroprobes during the immunostaining^22–32^. Conventional passive diffusion-based tissue clearing techniques, such as PEGASOS, can require up to 14 days to achieve optical clarity in rat spleen tissue^28^. Additionally, the performance of current whole tissue staining techniques, which predominantly rely on diffusion, often limits volumetric imaging to thin sections, with antibodies requiring days to diffuse mere millimeters into fixed tissues^33,34^. Therefore, alternative approaches have been explored that could reduce the processing time for tissue clearing and enhance the penetration of fluoroprobes. Current studies have explored the application of external forces, such as assisted perfusion^35^ and electric fields^34,36–40^. These have been shown to promote the diffusion of tissue clearing reagents into thick tissues^39^. Despite the notable benefits in applying these external forces, there is still a need for faster and more effective methods, particularly for dense collagenous tissues and heme-rich tissues. The existing perfusion-assisted tissue clearing method required 32 days to achieve optical clarity in heme-rich tissues such as mouse lung, heart, and liver^39^. Similarly, the application of electric fields necessitated 4 days to achieve optical clarity in adult rat liver and kidney tissues^34,36–40^. While these tissue clearing methods have demonstrated effectiveness in heme-rich tissues, there is currently no reported success in applying them to dense collagenous tissues. Moreover, applying high pressure to create convective flow risks damaging the tissue^41^. Similarly, electric fields can facilitate the ingress of staining molecules, but the need for strong currents to overcome resistance can deform molecular structures^42,43^. Low frequency ultrasound (LFU) sonication presents a promising alternative. Previous literature has shown that LFU enhances molecular permeability through sonoporation and cavitation effects^44–46^. Cavitation effects are produced by LFU and are characterized by the rapid formation and implosion of bubbles in a fluid. This leads to intense local pressure fluctuations which enhanced the vibrational effects that disrupt cell membranes and create transient openings—a phenomenon known as sonoporation^44,45^. Consequently, these mechanisms improve the distribution of reagents, facilitating improved penetration and distribution of various molecules, including small molecule drugs, proteins, and DNA, into tissues^44,45^. Based on these principles, we hypothesize that integrating LFU sonication into the tissue clearing and staining workflows could accelerate tissue clearing and enhance fluoroprobes diffusion.

**Fig. 1:**
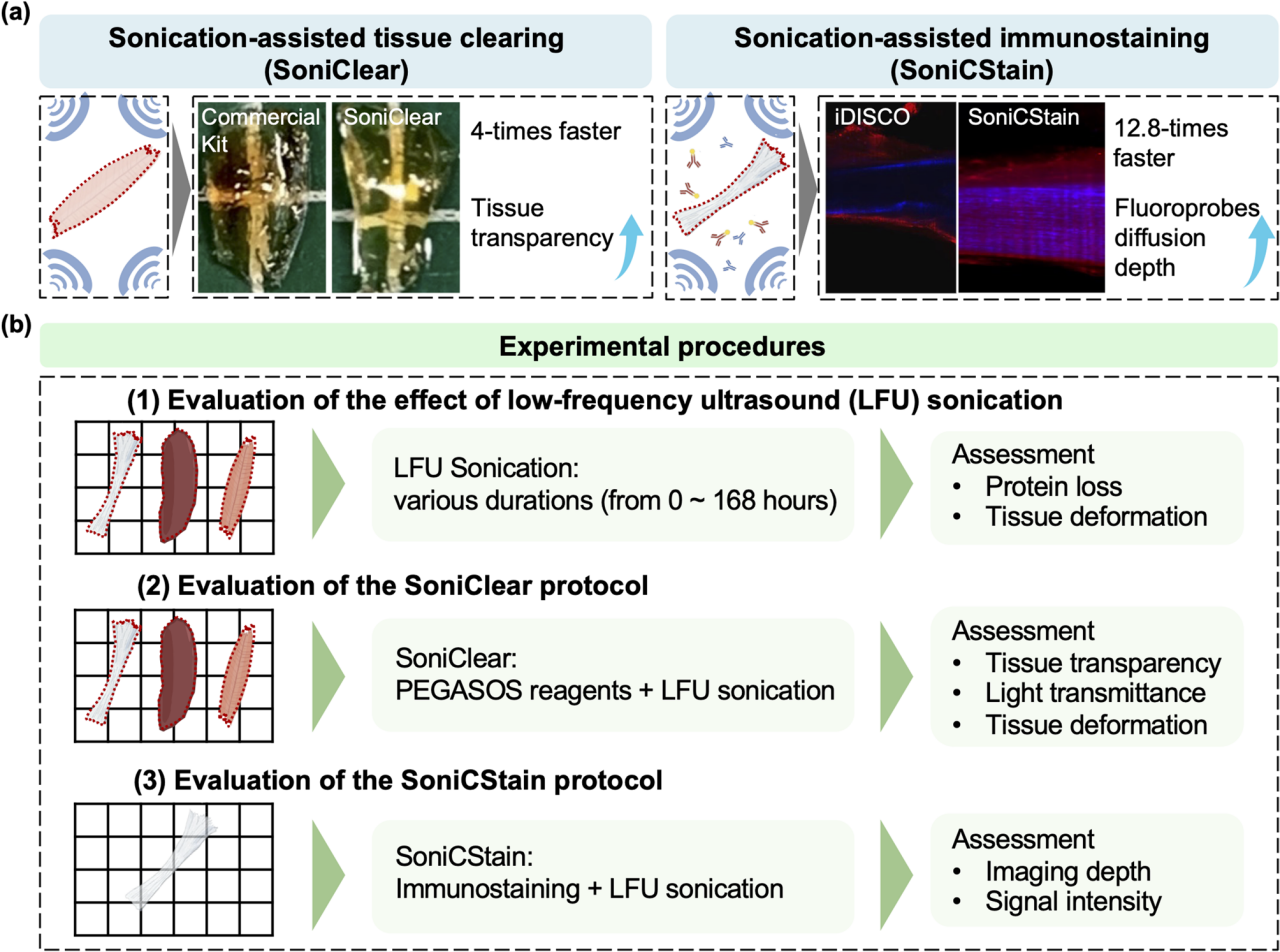
Research overview. **(a)** Schematic representation illustrating that in comparison to conventional tissue clearing techniques, SoniC/S exhibits several advantages, including increased time efficiency and enhanced diffusion depth of fluoroprobes. **(b)** Schematic representation of the experimental procedures undertaken in this study.

In this study, we introduce Sonication-Assisted Tissue Clearing and Immunofluorescent Staining (SoniC/S), a novel method that combines LFU sonication with a commercially available organic-solvent-based tissue clearing kit (PEGASOS, Leads Optics and Electro-physiology Limited, China) and iDISCO staining method (Fig. 1). First, the impact of different LFU sonication durations on tissue deformation and protein loss were evaluated in three tissue types: the soft tissue of mouse tibialis anterior (TA) muscle, the dense collagenous tissue of rat Achilles tendon, and a heme-rich tissue of mouse spleen. Subsequently, the tissue transparency, light transmittance, and the percentage of clearing achieved, were assessed in the three tissues using different SoniClear tissue clearing protocols. Following this, a SoniCStain immunofluorescent staining method was developed by integrating LFU sonication with the iDISCO staining protocol. Finally, the immunofluorescent intensity in rat Achilles tendon was quantified and compared to equivalent tissues processed using the conventional iDISCO staining method without LFU sonication.

## Method

### Collection of mouse TA muscle, spleen, and rat Achilles tendon tissues

Wild mice (C57BL/6J, 8–12 weeks old, male and female) and Sprague-Dawley rats (10–14 weeks old, male and female) were used in this study. All animals were housed on a 12 h light/dark cycle with free access to food and water in specific pathogen-free cages. The animal husbandry, animal care, and euthanasia procedures adhered to the guidelines outlined by The Chinese University of Hong Kong’s Animal Experimentation Ethics Committee and are approved under the following protocols: 21-170-MIS and 21-119-GRF. The study is also reported in accordance with the ARRIVE guidelines for reporting experiments involving animals (https://arriveguidelines.org). In this study, mouse TA muscle was used as a model for soft tissue, rat Achilles tendon was used as a representation of dense collagenous tissue, and mouse spleen was used as an example of heme-rich tissue. Rats and mice were euthanized via CO2 asphyxiation, utilizing a Quietek™ CO2 induction system (First vivo, USA). The exposure rate was calibrated to 50% of the chamber volume per minute, adhering to the manufacturer’s instructions and the standard operating procedures of the Laboratory Animal Science and Experimental Center (LASEC, The Chinese University of Hong Kong). Euthanasia duration was 10 min for rats and 8 min for mice. Confirmation of death was established through the absence of movement, heartbeat, respiration, and corneal reflex. TA muscles and spleens were extracted from mature wild type C57BL/6 mice aged between 8 and 12 weeks, encompassing both male and female specimens. Achilles tendons were obtained from male and female Sprague-Dawley rats aged 10 to 14 weeks. During the tissue dissections, adjacent muscle and adipose tissues were meticulously excised to prevent interference during imaging. The freshly obtained samples were gently washed in phosphate buffered saline (PBS; Santa Cruz Biotechnology, USA) and promptly submerged in a cold fixative solution containing 4% paraformaldehyde (PFA; Sigma-Aldrich, USA) for a duration of 24 h. Subsequently, the samples underwent three wash cycles with PBS at room temperature on a shaker, each lasting for an hour.

### Analysis of protein loss and tissue deformation in rat Achilles tendon, mouse TA muscle, and mouse spleen under various durations of LFU sonication

This study aimed to integrate LFU sonication with the established PEGASOS tissue clearing method. Before developing the SoniC/S method, a protein loss analysis and quantitative deformation analysis were performed. These analyses were conducted to assess the extent of protein loss and the degree of tissue deformation induced by external forces during LFU sonication. Fixed Achilles tendons from rats, TA muscles and spleens from mice were immersed in PBS for this evaluation. Three different conditions were tested: passive immersion, gentle shaking and sonification. In the passive immersion group, specimens were left to incubate at 37 °C without external force. These samples were designated as the control group. To emulate the conditions of the PEGASOS protocol, samples were maintained at 37 °C with gentle shaking at 1000 rpm. In the sonication group, representing the SoniClear environment, samples were subjected to 40 kHz LFU at 37 °C with low intensity (0.370 W/cm^2^; Ultrasonic Cleaner CH10BM, ULTRASONIC CLEANER, China). At designated time points, it is 0, 1, 3, 6, 12, 24, 72, and 168 h, PBS were collected, and the degree of protein loss under the mentioned conditions was assessed using a bicinchoninic acid (BCA) protein assay kit (Thermo Fisher Scientific, USA) according to the manufacturer’s protocol. The protein levels at each time point were normalized based on the initial sample weights at 0 h. Moreover, tissue samples were imaged and quantitatively assessed at intervals of 0, 1, 3, 6, 12, 24, 72, and 168 h to determine the percentage change in sample size. The quantitative deformation analysis was conducted by measuring alterations in sample dimensions. The contours of tissue samples were outlined using the “polygon-selections” tool within the Fiji software following image thresholding^47–49^. Manual alignment of the outlines was meticulously carried out. The degree of deformation was quantified by calculating the absolute variance between tissue size measured at the designated time points and tissue sizes at 0 h as a percentage of the initial sample size^14,47,48^.

## Procedures of tissue clearing using PEGASOS and SoniClear methods

The established PEGASOS method was used as the control tissue clearing method in this study. Therefore, the intact mouse TA muscle, mouse spleen, and rat Achilles tendon were subjected to optical clearing employing the Leads Tissue Clearing Solution Kit-PEGASOS (Leads Optics and Electro-physiology Limited, China). In short, the procedure involved treating the samples with Quadrol decolorization solution (solution 2 from the kit) for 48 h on a shaker at 37 °C. Subsequently, the samples underwent immersion in a gradient of delipidating solutions (solutions 3–5 from the kit) on a 37 °C shaker for 48 h each, followed by treatment with tB-PEG dehydration solution (solution 6) for 24 h on a shaker at 37 °C. Finally, the samples were immersed in BB-PEG refractive index matching medium (solution 7) for 48 h on a 37 °C shaker until achieving transparency. The samples were then stored in the refractive index matching medium (solution 7) at room temperature. As a control, mouse TA muscle, mouse spleen, and rat Achilles tendon were incubated at PBS at 37 °C for the same total processing time as the PEGASOS method. Details regarding the processing time, temperature conditions, and shaking speed for the immersion-based tissue clearing process are provided in the supplementary information (Supplementary Table 1).

Subsequently, the SoniClear protocols were developed by refining the original PEGASOS method to reduce the processing time. In essence, fixed mouse TA muscle, mouse spleen, and rat Achilles tendon were exposed to the PEGASOS tissue clearing reagents, including those for decolourization, delipidation, dehydration and refractive index matching (Leads Tissue Clearing Solution Kit-PEGASOS, Leads Optics and Electro-physiology Limited, China), under LFU sonication at 40 kHz. SoniClear protocol variations included: (1) SoniClear-1 (1 h for each step); (2) SoniClear-2 (3 h for each step); (3) SoniClear-3 (6 h for each step); (4) SoniClear-4 (12 h for each step). Note that for spleen SoniClear protocols, an additional 3 h of pre-incubation is required for removal of superficial pigments prior to tissue clearing. The specifics of the time schedule, temperature settings, and reagents utilized for the SoniClear protocols are outlined in the supplementary information (Supplementary Tables 2 & 3).

## Quantitative deformation analysis before and after SoniClear

To assess tissue dimensional changes following the completion of the tissue clearing process, the quantitative deformation analysis of rat Achilles tendon, mouse TA muscle, and spleen before and after SoniClear was conducted. The contours of tissue samples were outlined using the “polygon-selections” tool within the Fiji software following image thresholding^47,48^. Manual alignment of the outlines was meticulously carried out. The degree of deformation was quantified by calculating the absolute variance between post-processing and pre- processing tissue sizes as a percentage of the initial sample size^14,47,48^. Linear expansion was calculated by normalizing the area of the cleared tissue sample against its corresponding pre-cleared sample, followed by the calculation of the square root of this ratio^14,47,48^.

## Light transmittance analysis before and after SoniClear

To further assess the efficacy of the SoniClear protocol, an essential parameter under consideration is light transmittance. To quantify transmittance, the tissue samples were sliced into cubes with dimensions of 1 mm × 1 mm × 1 mm and were subsequently analyzed using a spectrophotometer (Multiskan GO, Thermo Fisher Scientific, USA)^14^. Briefly, the tissue samples pre- and post-clearing were inserted into a cuvette in such a way that light passed through the samples along the anterior-posterior axis, and the transmittance was evaluated across the UV-Vis-NIR spectrum (ranging from 350 to 1000 nm). The baseline measurement was taken with a clearing reagent (Solution 7, Leads Tissue Clearing Solution Kit-PEGASOS, China) in the absence of a sample, and the transmittance of the sample was then normalized to this baseline value^25^.

## Tissue transparency analysis after SoniClear

For quantification of transparency, a USAF Resolution Target chart was used and values (line-pairs per mm) were calculated to represent levels of transparency. The USAF target chart contains several elements. Each element consists of a group of three bars of equal width with the spacing between the bars being the same width as well^50^. Each element has a corresponding number, it is the line-pairs per mm. The tissue sample was positioned over the USAF target chart. An imaginary line was delineated across the region of interest, as depicted in Figure S1. Gray values along this line were extracted utilizing the “Plot Profile” function within ImageJ software (Supplementary Fig. 1). If the gray value of wave valley (G_min_) is less than or equal to 8/π^2^ times to that of wave peak (G_max_), the region of interest will be identifiable^51,52^. In this way, the last identifiable element was found and their corresponding number (line pairs per mm) was defined as level of transparency.

### Whole-tissue immunofluorescent staining

Whole-tissue immunofluorescent staining was performed utilizing rat Achilles tendon as a model. Following the delipidation stage during SoniClear-3, tissue samples underwent blocking and immunofluorescent staining as described below.

A modified version of the conventional whole-tissue immunofluorescent staining protocol used in iDISCO and PEGASOS tissue clearing methods was used as the control group^28,29^. In short, first the tissue samples were immersed in a pretreatment solution consisting of 0.6 M glycine (1610718, BIO-RAD, USA), 20% DMSO (D4540-1 L, Sigma-Aldrich, USA), 0.2% Triton X-100 (9002-93-1, Sigma-Aldrich, USA), and 6% Donkey serum (D9663, Sigma-Aldrich, USA) in 1xPBS. This blocking and permeabilization step were carried out over 24 h at 4 °C with shaking at 220 rpm. Subsequently, the samples were treated with a primary antibody, anti-COL-1 (1:500; ab90395, AbCam, United Kingdom), diluted in the pretreatment buffer for 3 days at 4 °C under the same agitation conditions. Following this, the samples underwent three washes in PBS for 1 h each at 4 °C under agitation at 220 rpm. Subsequently, the samples will be exposed to a secondary antibody diluted in the pretreatment buffer, namely Alexa Fluor 647 donkey anti-mouse IgG (1:500; A31571, Invitrogen, USA). Finally, the samples were washed again and counterstained with diluted Hoechst 33342 nuclear stain (1:1000; 62249, Thermo Fisher Scientific, USA).

To shorten the duration of the immunofluorescent staining process and enhance deep tissue staining, the SoniCStain protocols were developed. Tissue samples post-delipidation were initially blocked and permeabilized using the pretreatment solution under low-frequency ultrasound sonication at 40 kHz for 3 h. The samples were then subjected to the primary antibody (anti-COL-1; 1:500; ab90395, AbCam, United Kingdom) diluted in the pretreatment buffer under low-frequency ultrasound sonication at 40 kHz with varying duration as specified in Supplementary Table 3. After three rounds of wash in PBS at 4 °C under shaking at 220 rpm for 1 h each, the samples were exposed to the secondary antibodies and nuclear stain. The secondary antibodies and nuclear stain, namely Alexa Fluor 647 donkey anti-mouse IgG (1:500; A31571, Invitrogen, USA) and Hoechst 33342 nuclear stain (1:1000; 62249, Thermo Fisher Scientific, USA), were diluted in the pretreatment buffer and staining occurred under low-frequency ultrasound sonication at 40 kHz with varying duration as specified in Supplementary Table 3. The specific processing time and temperature settings for the SoniCStain protocols can be found in Supplementary Information (Supplementary Table 3).

## Whole-tissue immunofluorescent imaging and data processing

Fluorescent signals emanating from optically cleared tissue specimens were captured employing a light sheet microscopy system (LiToneXL, Light Innovation Technology, China) featuring an XLFLUOR 4x Apochromatic Long Working Distance (LWD) objective lens possessing a numerical aperture (NA) of 0.28 and a working distance of 20 mm. This system was complemented by the Solar 2.0 Multi-channel Laser Unit (Light Innovation Technology, China). Illumination of thin light sheets was orchestrated from both the left and right sides of the sample through the utilization of the 4-sided Line Bessel Sheet generation module, culminating in the acquisition of a composite image. During the light sheet imaging process, laser power was set to 10%, with an exposure duration of 250 milliseconds. Z-stacks were obtained utilizing 358 nm and 647 nm laser wavelengths to excite Hoechst 33342 and Alexa Fluor 647, respectively, with a Z-interval of 10 μm.

All primary image data were acquired and preserved in a lossless TIFF format of 16-bit and 8-bit. Subsequently, the 16-bit images underwent seamless integration using the proprietary LitScan 2.0 software (Light Innovation Technology, China) and were further transmuted to the Imaris format. 3D image construction was conducted using Imaris 9 software (Bitplane, Switzerland). To evaluate the efficiency of the SoniCStain methods and the fluorescence intensity in the Achilles tendon samples, the 8-bit image stacks (Z-step size: 10 μm; Depth: 1000 μm) were first imported in FIJI. Afterward, fluorescence intensity values were measured using Fiji according to the prior protocol^49^. Fluorescence intensity was measured at every Z-step. The fluorescence intensity of Achilles sample without immunofluorescent staining was used as a control, and the fluorescence intensity value was normalized as 1.00. The relative fluorescence intensity was shown as the ratio of fluorescence intensity of the experimental group over time to fluorescence intensity of the control group.

### Statistical analysis

Statistical analyses were performed using GraphPad Prism software (Version 10.0.0, USA). All experiments were performed with 3–4 per group and quantitative data were presented as mean ± standard deviation (mean ± s.d.) as the figure legend described. Sample sizes are noted in figure legends. Statistical significance was determined using the one-way ANOVA for multiple comparisons. *: *p* < 0.05; **: *p* < 0.01; ***: *p* < 0.001; and ****: *p* < 0.0001.

## Results

### Impact of LFU sonication on tissue deformation and protein loss

LFU sonication has proven effective in enhancing cell membrane permeability and facilitating molecular diffusion^44,45^. However, it is essential to optimize the duration of LFU sonication to preserve sample integrity^53–56^. Therefore, tissue deformation and protein loss were evaluated in rat Achilles tendons, mouse TA muscles, and mouse spleens, subjected to either passive immersion (control), gentle shaking (PEGASOS protocol) or LFU sonification at the specified time intervals.

**Fig. 2:**
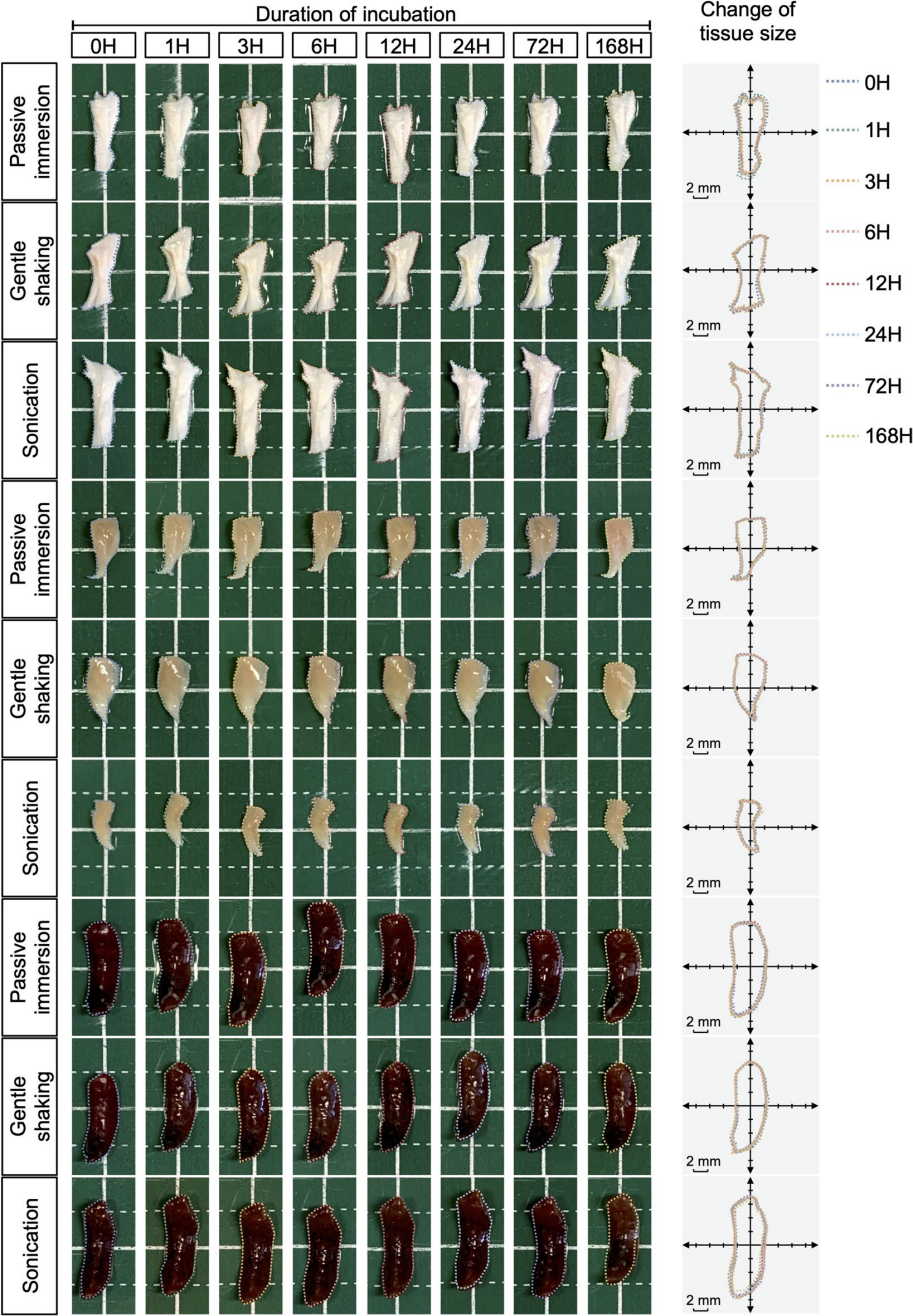
**Macroscopic images of rat Achilles tendon**,** mouse TA muscle**,** and mouse spleen subjected to passive immersion**,** gentle shaking**,** and LFU sonication**. No significant changes in tissue size were observed across all groups over the 168-hour incubation period. Grid size: 5 mm × 5 mm. *n* = 3–4 for each group.

**Fig. 3:**
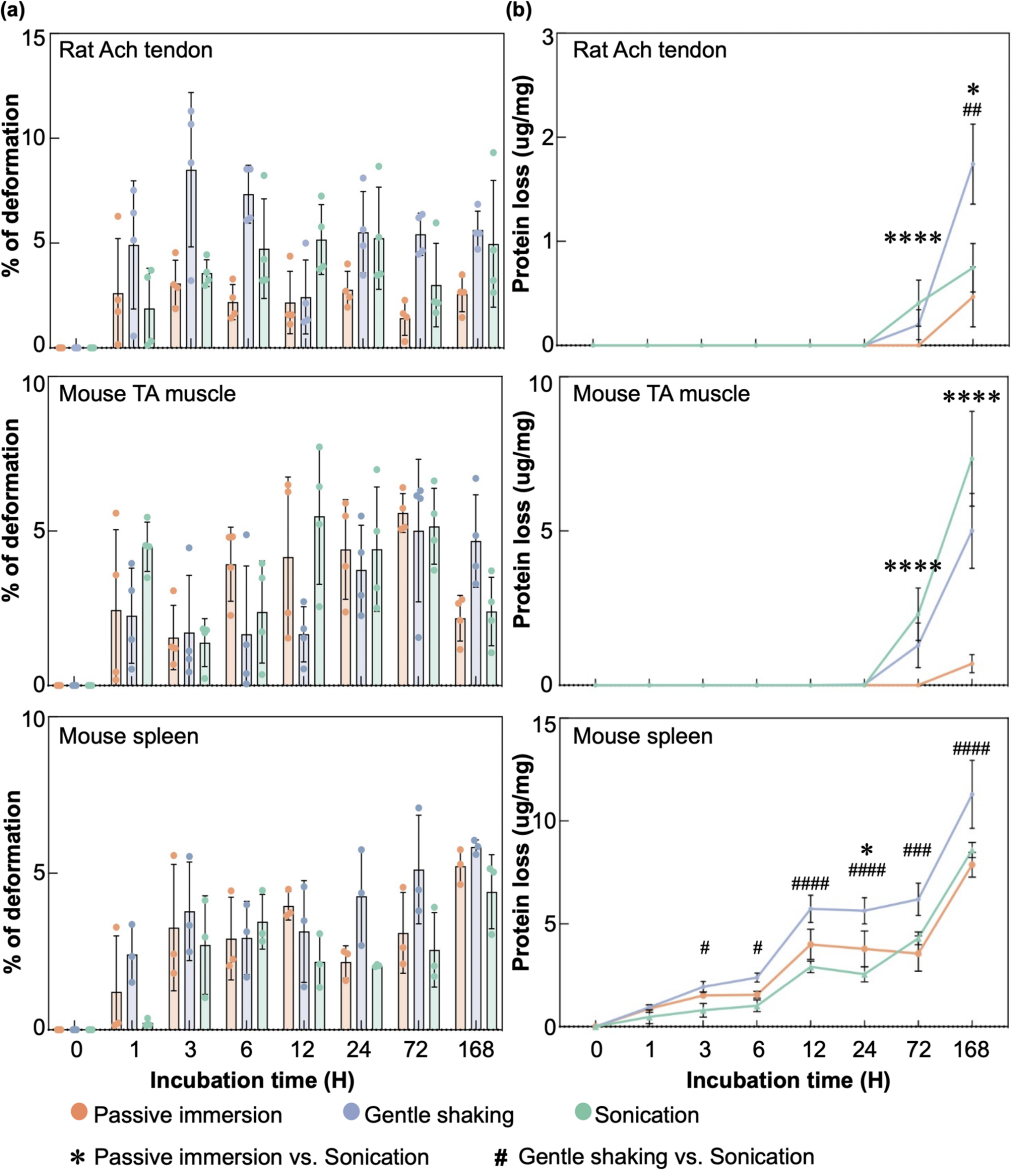
**Assessment of sonication-induced tissue deformation and protein loss in rat Achilles tendon**,** mouse TA muscle**,** and mouse spleen**. **(a)** Quantitative analysis of tissue deformation in Achilles tendon, TA muscle, and spleen subjected to passive immersion, gentle shaking, and LFU sonication in PBS at 37 °C for 168 h revealed a comparable percentage of deformation between the sonication group and the passive immersion control in all tissues (*n* = 3–4 for each group). **(b)** Evaluation of protein loss in Achilles tendon, TA muscle, and spleen under the same experimental conditions indicated that protein loss in the sonication group after 168 h was comparable to the gentle shaking group and greater than the passive immersion group for both Achilles tendon and TA muscle. In contrast, protein loss in the spleen under sonication for 168 h was similar to that of the passive immersion group (*n* = 3–4 for each group). mean ± s.d.; *, ^#^, *p* < 0.05; **, ^##^, *p* < 0.01; ***, ^###,^ *p* < 0.001; ****, ^####^, *p* < 0.0001.

Macroscopic images showed that the change in tissue size following an incubation period of 168 h in rat Achilles tendon, mouse TA muscle, and mouse spleen subjected to LFU sonication was comparable to the changes observed in these tissues processed through passive immersion and gentle shaking at each time point (Fig. 2). Quantitative analysis revealed that at the 6-hour time point, the percentage of tissue deformation was comparable between the sonication group and the passive immersion control: Achilles tendons exhibited deformation percentages of 4.74 ± 2.38% versus 2.19 ± 0.84%, TA muscles showed 2.85 ± 2.23% versus 5.44 ± 3.19%, and spleens presented 3.45 ± 0.88% versus 2.91 ± 1.32%. Similarly, the percentage of tissue deformation was comparable between the sonication group and the gentle shaking group (Fig. 3a). Upon extending the incubation to 168 h, a trend toward increased tissue deformation was observed in both Achilles tendons, TA muscles, and spleens, although the percentage of deformation remained comparable between the sonication group and the passive immersion control. Notably, the deformation percentages in the sonication group for the three types of tissue were all lower than those in the gentle shaking group (Fig. 3a). These findings suggest that LFU sonication at 40 kHz for 168 h has a minimum impact on tissue deformation.

Subsequently, protein loss associated with LFU sonication was quantified in each tissue by comparing the extent of protein loss in the sonication group to the passive immersion group and the gentle shaking group at different timepoints. In both TA muscles and Achilles tendons, protein loss was negligible across all conditions at the 24 h timepoint. In spleen, a trend towards increased protein loss was observed. However, the normalized protein loss under sonication during the first 24 h was not statistically different from the passive immersion group. The protein loss at the 24 h timepoint in the spleen sonication group was 2.55 ± 0.37 µg/mg, while the spleen passive immersion group exhibited a loss of 3.78 ± 0.67 µg/mg (*p* = 0.1931). Conversely, spleen protein loss under sonication was significantly lower than observed in the gentle shaking group (5.64 ± 0.63 µg/mg; *p* < 0.0001). At 72 h, a slight increase in protein loss was observed in both Achilles tendons (0.41 ± 0.22 µg/mg), TA muscles (2.30 ± 0.85 µg/mg), and spleen (4.30 ± 0.31 µg/mg) after sonification compared to the passive immersion group (Achilles tendon: 0.00 ± 0.00 µg/mg; TA muscle: 0.00 ± 0.00 µg/mg; spleen: 3.56 ± 0.86 µg/mg), although these values remained comparable to those in the gentle shaking group (Achilles tendon: 0.20 ± 0.14 µg/mg; TA muscle: 1.29 ± 0.72 µg/mg; spleen: 6.19 ± 0.78 µg/mg). At 168 h, a further increase in protein loss was observed in Achilles tendons (0.75 ± 0.23 µg/mg) and TA muscles (7.34 ± 1.53 µg/mg) after sonification compared to the passive immersion group (Achilles tendon: 0.47 ± 0.29 µg/mg; TA muscle: 0.67 ± 0.29 µg/mg), although these values remained comparable to those in the gentle shaking group (Achilles tendon: 1.74 ± 0.38 µg/mg; TA muscle: 5.01 ± 1.21 µg/mg). For spleen tissues, the normalized amount of protein loss under sonication for 168 h (8.59 ± 0.37 µg/mg) did not differ statistically from the passive immersion group (7.88 ± 0.60 µg/mg, *p* = 0.2761). Conversely, protein loss under sonication was significantly lower than observed in the gentle shaking group (*p* < 0.0001) (Fig. 3b). In short, LFU sonication for brief durations has negligible effects on protein loss. For extended processing times, protein loss in Achilles tendons and TA muscles under sonication conditions is comparable to that observed with gentle shaking. Notably, spleen samples exhibited protein loss levels comparable to those in the passive immersion group.

Collectively, these findings indicate that LFU sonication at 40 kHz for a duration of up to 168 h resulted in tissue deformation and protein loss comparable to the passive immersion control group.

### Effect of SoniClear on transparency and processing time in mouse TA muscle

**Fig. 4:**
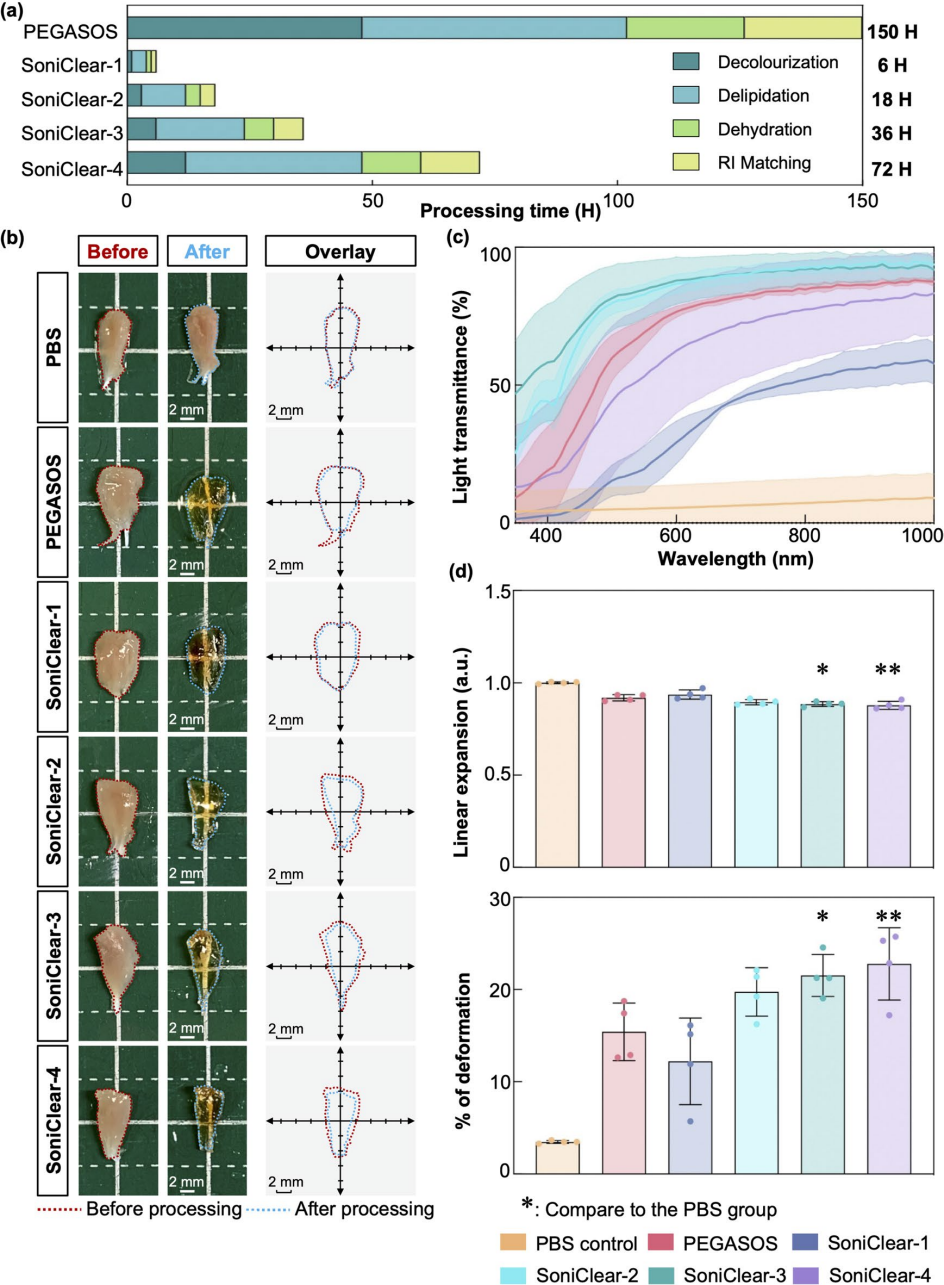
**Evaluation of tissue transparency**,** deformation**,** and light transmittance in mouse TA muscle following processing with PEGASOS and SoniClear methods**. **(a)** Schematic representation of processing durations for the PEGASOS and SoniClear. **(b)** Comparative macroscopic images of mouse TA muscle before and after processing in different experimental groups demonstrate that SoniClear-processed samples achieved tissue transparency comparable to that of the commercial PEGASOS method. Overlay images illustrate the contours of the samples, demonstrating that mouse TA muscle processed with SoniClear retained its original dimensions (*n* = 4 for each group). **(c)** Light transmission analysis indicated that SoniClear-2- and SoniClear-3-processed samples exhibited higher light transmittance compared to those processed with the PEGASOS method (*n* = 3–4 for each group). **(d)** Linear expansion and deformation analyses revealed no statistically significant alterations in linear expansion or percentage deformation for SoniClear methods, except for SoniClear-3 and -4, when compared to the PBS control and PEGASOS groups (*n* = 4 for each group). Data in (c) and (d) are expressed as mean ± s.d.; *, *p* < 0.05; **, *p* < 0.001.

To assess the efficacy of SoniClear in tissue clearing, a comparative analysis against the conventional PEGASOS protocol was conducted using fixed mouse TA muscle samples. In contrast to the conventional protocol, SoniClear employed the PEGASOS kit in combination with LFU sonication for designated intervals during each processing step (Fig. 4a). Control samples were immersed in 1x PBS at 37 °C for a total of 150 h without external manipulation.

Visual assessment indicated that even the shortest processing time with SoniClear resulted in a transparency level comparable to that achieved via the PEGASOS tissue clearing kit, while maintaining minimal changes in tissue size (Fig. 4b; Supplementary Fig. 2a). Quantitative analysis of tissue transparency validated the enhanced clearing performance of SoniClear. Specifically, SoniClear-1 demonstrated a 1.5-fold increase in tissue transparency while reducing total processing time by a factor of 25 compared to PEGASOS. Moreover, as processing time was extended to 18, 36, and 72 h, the tissue transparency of samples processed with SoniClear-2, -3, and  -4 increased by factors of 1.9, 2.1, and 2.3, respectively, relative to samples processed with PEGASOS (Supplementary Table 6). Besides tissue transparency, light transmittance is also an indicator to analyze the clearing capacity. Light transmittance measurements of mouse TA muscle tissue blocks across the UV-Vis-NIR spectrum revealed that SoniClear-2 and SoniClear-3 exhibited superior clearing capabilities compared to PEGASOS. In contrast, samples processed with SoniClear-1 and SoniClear-4 demonstrated reduced light transmittance relative to the PEGASOS group. For example, SoniClear-2 and SoniClear-3 exhibited higher light transmittance at various wavelengths compared to PEGASOS: at 400 nm (PEGASOS: 19.03 ± 15.91%; SoniClear-2: 44.13 ± 7.82%; SoniClear-3: 58.17 ± 21.22%), 600 nm (PEGASOS: 76.56 ± 2.00%; SoniClear-2: 86.87 ± 3.36%; SoniClear-3: 87.94 ± 8.60%), 800 nm (PEGASOS: 84.69 ± 1.93%; SoniClear-2: 92.51 ± 2.15%; SoniClear-3: 92.02 ± 5.79%), and 1000 nm (PEGASOS: 87.54 ± 1.61%; SoniClear-2: 93.70 ± 1.54%; SoniClear-3: 91.81 ± 4.32%) (Fig. 4c; Supplementary Fig. 2b). These results indicate that SoniClear protocols, particularly SoniClear-2 and -3, not only facilitate comparable tissue transparency but also exhibit superior light transmittance in contrast to the PEGASOS, all while significantly reducing processing time.

Additionally, the degree of deformation in TA muscle samples was evaluated across the PBS control, PEGASOS, and SoniClear groups. Image analysis revealed that samples treated with both PEGASOS and SoniClear exhibited a reduced tissue size relative to the PBS control (Fig. 4d). Quantitative measurements of linear expansion indicated that both PEGASOS-processed (0.92 ± 4.32 a.u.) and SoniClear-processed muscles (SoniClear-1: 0.94 ± 0.02 a.u.; SoniClear-2: 0.90 ± 0.01 a.u.; SoniClear-3: 0.89 ± 0.01 a.u.; SoniClear-4: 0.88 ± 0.02 a.u.) yielded values below 1.0 a.u., signifying tissue shrinkage (Fig. 4d). Notably, with the exception of SoniClear-3 and -4, the observed changes in linear expansion and percentage deformation were not statistically significant compared to control (Fig. 4d).

Collectively, SoniClear is comparable to the PEGASOS protocol regarding tissue transparency, light transmittance, and the degree of deformation in mouse TA muscle, while significantly reducing the processing time.

### Effect of SoniClear on transparency and processing time in rat Achilles tendon

Despite the development of various tissue clearing protocols, their efficacy in dense collagenous tissues, such as tendons, remains inadequately explored. The dense collagenous network, elevated viscoelastic properties, and extracellular matrix of these tissues are hypothesized to obstruct the diffusion and infiltration of clearing agents^1,57–60^. To address this issue, the SoniClear protocols were applied on fixed rat Achilles tendon.

**Fig. 5:**
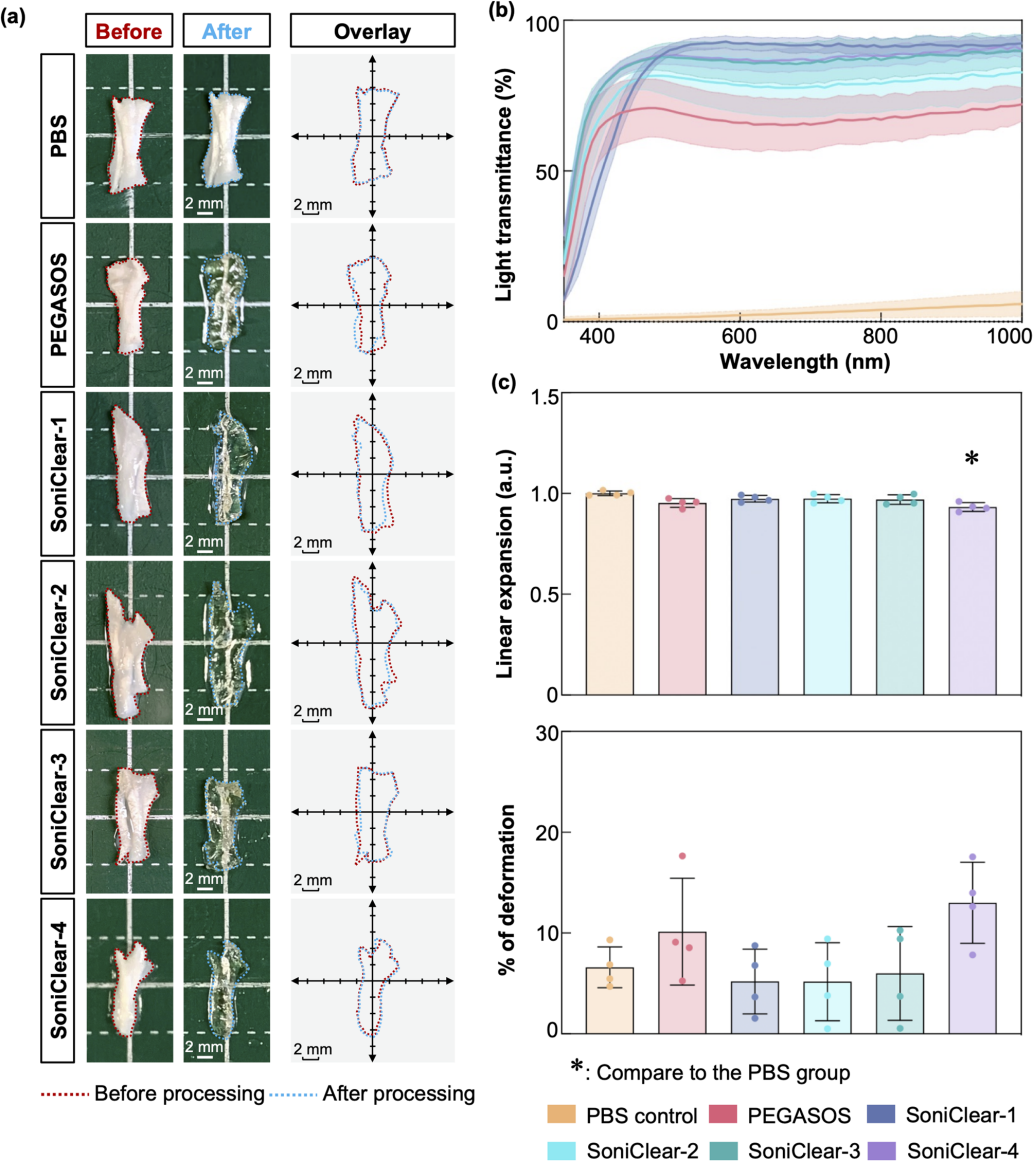
**Evaluation of tissue transparency**,** deformation**,** and light transmittance in rat Achilles tendon following processing with PEGASOS and SoniClear methods**. **(a)** Comparative macroscopic images of rat Achilles tendon before and after processing in different experimental groups, demonstrating that SoniClear-processed samples achieved tissue transparency comparable to that of the commercial PEGASOS method. Overlay images illustrate the contours of the samples, demonstrating that rat Achilles tendons processed with SoniClear retained its original dimensions (*n* = 4 for each group). **(b)** Light transmittance analysis showed that SoniClear-processed samples exhibited higher light transmittance compared to the PEGASOS method (*n* = 3–4 for each group). **(c)** Linear expansion and deformation analyses of rat Achilles tendons revealed that the SoniClear methods demonstrated statistically insignificant changes in linear expansion and percentage deformation, except for SoniClear-4, when compared to the PBS control and PEGASOS groups (*n* = 4 for each group). Data in (b) and (c) are expressed as mean ± s.d. *, *p* < 0.05.

Macroscopic evaluations demonstrated that the SoniClear protocol effectively rendered the rat Achilles tendon transparent at the whole tissue level (Fig. 5a; Supplementary Fig. 3a). Notably, the transparency achieved in SoniClear-processed samples was comparable to that of PEGASOS-processed samples. Quantitative analysis confirmed that the tissue transparency post-SoniClear processing was akin to that of the PEGASOS method. Tissue transparency measurements demonstrated PEGASOS at 32.00 line-pairs per mm and SoniClear-1 at 31.13 Line-pairs per mm. An upward trend in tissue transparency was observed when processing time of SoniClear protocol was extended to 72 h with SoniClear-4, yielding a measurement of 44.33 line-pairs per mm (Supplementary Table 6). Light transmittance measurements further corroborated the enhanced light transmittance of SoniClear-processed tendon samples. Each variant of the SoniClear method exhibited superior light transmittance compared to the use of PEGASOS kit. Among the four different SoniClear protocols, SoniClear-3 demonstrated the highest value of light transmittance when compared to the PEGASOS. Comparative analysis revealed that SoniClear-3 outperformed PEGASOS at multiple wavelengths: 400 nm (PEGASOS: 62.85 ± 8.10%; SoniClear-3: 76.45 ± 3.78%), 600 nm (PEGASOS: 65.52 ± 6.33%; SoniClear-3: 86.08 ± 6.51%), 800 nm (PEGASOS: 66.95 ± 5.23%; SoniClear-3: 86.85 ± 6.53%), and 1000 nm (PEGASOS: 72.07 ± 3.94%; SoniClear-3: 89.83 ± 5.39%) (Fig. 5b; Supplementary Fig. 3b). These findings underscore the superior efficacy of the SoniClear protocols in achieving enhanced tissue transparency and light transmittance in rat Achilles tendons.

Post-clearing visual assessments indicated that SoniClear-processed tendons retained their original dimensions, exhibiting no significant expansion or shrinkage (Fig. 5a). Quantitatively, with the exception of samples processed with SoniClear-4, the linear expansion of tendons subjected to SoniClear processing was lower than that observed with PEGASOS and comparable to that of the PBS control group (PBS control: 1.00 ± 0.01 a.u.; SoniClear-1: 0.97 ± 0.02 a.u.; SoniClear-2: 0.97 ± 0.02 a.u.; SoniClear-3: 0.97 ± 0.02 a.u.; SoniClear-4: 0.93 ± 0.02 a.u.) (Fig. 5c).

Collectively, SoniClear demonstrated effects analogous to the PEGASOS kit with respect to tissue transparency, light transmittance, and the extent of deformation in rat Achilles tendon specimens, while markedly decreasing processing time.

### Effect of SoniClear on transparency and processing time in spleen

Following the successful application of SoniClear in muscle and tendon tissues, its efficacy in clearing pigment-rich tissues was evaluated. The presence of highly pigmented components poses significant challenges for tissue clearing due to increased light absorption and light scattering, often requiring extended decolorization periods, leading to prolonged processing times^52,61^. To address these issues, our study adapted SoniClear protocols for the heme-rich mouse spleen. The mouse spleen samples were initially incubated in a decolorization reagent for three hours at room temperature to facilitate the removal of superficial pigment. Subsequently, the efficacy of SoniClear was evaluated and compared to the PEGASOS tissue clearing kit and PBS control.

**Fig. 6:**
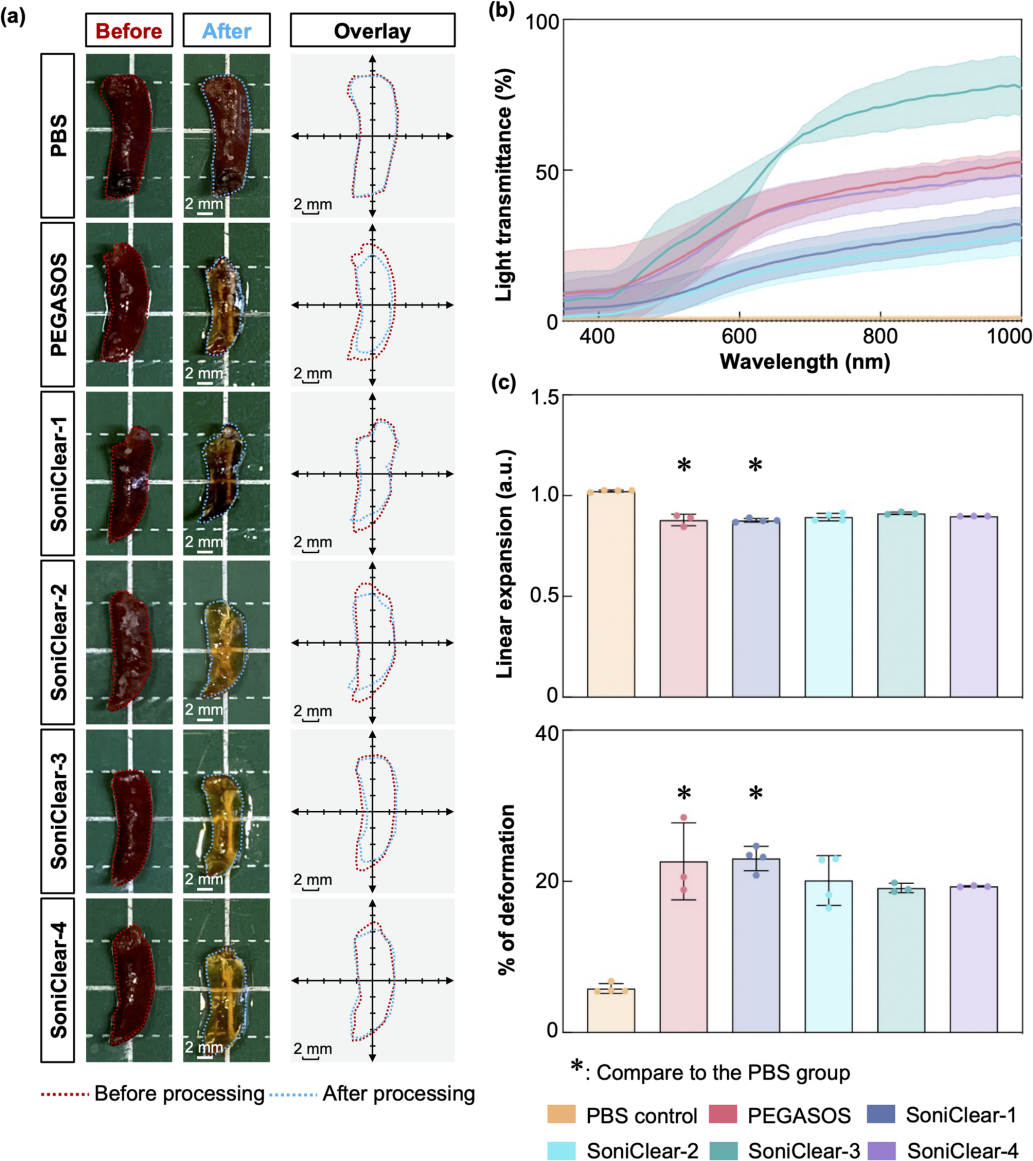
**Evaluation of tissue transparency**,** deformation**,** and light transmittance in mouse spleen following processing with PEGASOS and SoniClear methods**. **(a)** Comparative macroscopic images of mouse spleen before and after processing in different experimental groups. Except for SoniClear-1, SoniClear-processed samples achieved higher tissue transparency than the PEGASOS-processed samples. Overlay images illustrate the contours of the sample, highlighting that mouse spleens processed with SoniClear retained their original dimensions without significant tissue shrinkage when compared to the PEGASOS-processed samples (*n* = 3–4 for each group). **(b)** Light transmittance analysis showed that SoniClear-3-processed samples exhibited higher light transmittance compared to the PEGASOS-processed samples (*n* = 3–4 for each group). **(c)** Linear expansion and deformation analyses of mouse spleens revealed that SoniClear-3- and -4-processed samples demonstrated statistically insignificant changes in linear expansion and percentage deformation when compared to the PBS control and PEGASOS groups (*n* = 3–4 for each group). Data in (b) and (c) are expressed as mean ± s.d. *, *p* < 0.05.

Post-clearing assessments demonstrated that tissues processed with SoniClear exhibited transparency levels comparable to those obtained via PEGASOS. Notably, macroscopic imaging indicated that spleen samples processed with SoniClear-2, -3, and -4 achieved enhanced overall transparency relative to the use of PEGASOS kit (Fig. 6a; Supplementary Fig. 4a). Quantitative analyses indicated that SoniClear protocols-2, -3, and -4 resulted in 1.47-fold, 1.56-fold, and 1.47-fold increases in tissue transparency, respectively, compared to PEGASOS, while concurrently reducing total processing times by 8.33-fold, 4.17-fold, and 2.08-fold. In contrast, the tissue transparency in samples processed with SoniClear-1 was lower than those processed with PEGASOS (Supplementary Table 6). Light transmittance measurements indicated that mouse spleen samples processed with SoniClear-1 and SoniClear-2 exhibited lower light transmittance compared to those treated with PEGASOS. In contrast, samples processed with SoniClear-3 demonstrated higher light transmittance, while SoniClear-4 achieved light transmittance comparable to that of the PEGASOS-treated samples. For instance, SoniClear-3 demonstrating superior transmittance across multiple wavelengths: 400 nm (PEGASOS: 9.88 ± 14.21%; SoniClear-3: 7.10 ± 9.13%), 600 nm (PEGASOS: 32.18 ± 7.66%; SoniClear-3: 40.40 ± 9.14%), 800 nm (PEGASOS: 45.56 ± 4.72%; SoniClear-3: 40.87 ± 8.30%), and 1000 nm (PEGASOS: 52.75 ± 3.75%; SoniClear-3: 77.26 ± 9.20%) (Fig. 6b; Supplementary Fig. 4b). These results suggest that SoniClear, particularly the SoniClear-3 protocol, enhances both tissue transparency and Light transmittance while significantly reducing the total processing time to 36 h.

Additionally, the tissue deformation was assessed, with both SoniClear- and PEGASOS-processed spleens showing a reduction in tissue size compared to the PBS control group. However, SoniClear-processed spleens exhibited less deformation than those processed with PEGASOS, as evidenced by macroscopic imaging (Fig. 6a) and quantitative analysis: PEGASOS (22.65 ± 5.10%); SoniClear-1 (23.03 ± 1.62%); SoniClear-2 (20.12 ± 3.29%); SoniClear-3 (19.13 ± 0.61%); SoniClear-4 (19.34 ± 0.08%) (Fig. 6c). These findings indicate that while some deformation is present relative to the PBS-processed control, SoniClear protocols facilitate a reduction in tissue deformation compared to the commercial PEGASOS kit.

Collectively, SoniClear demonstrated effects that were comparable to those of the commercial PEGASOS kit in terms of tissue transparency, light transmittance, and the extent of deformation in mouse spleen.

### Effect of SoniCStain on antibody penetration and processing time in rat Achilles tendon

Conventional whole-tissue immunofluorescent protocols are often labor-intensive, spanning several days to weeks^62^, and suffer from inadequate antibody penetration, particularly in dense collagenous tissues^39,63^. Peripheral riming—characterized by fluoroprobe accumulation at the tissue periphery—further restricts diffusion into central regions^62^. To address these challenges and expedite antibody delivery, the SoniCStain method was introduced, which integrates LFU sonication during the immunofluorescent staining process. Rat Achilles tendon samples were subjected to optical clearing via the SoniClear-3 protocol, followed by staining using a modified conventional protocol (passive immersion group)^29^ or the SoniCStain method. Finally, non-stained samples serve as controls for signal intensity normalization.

**Fig. 7:**
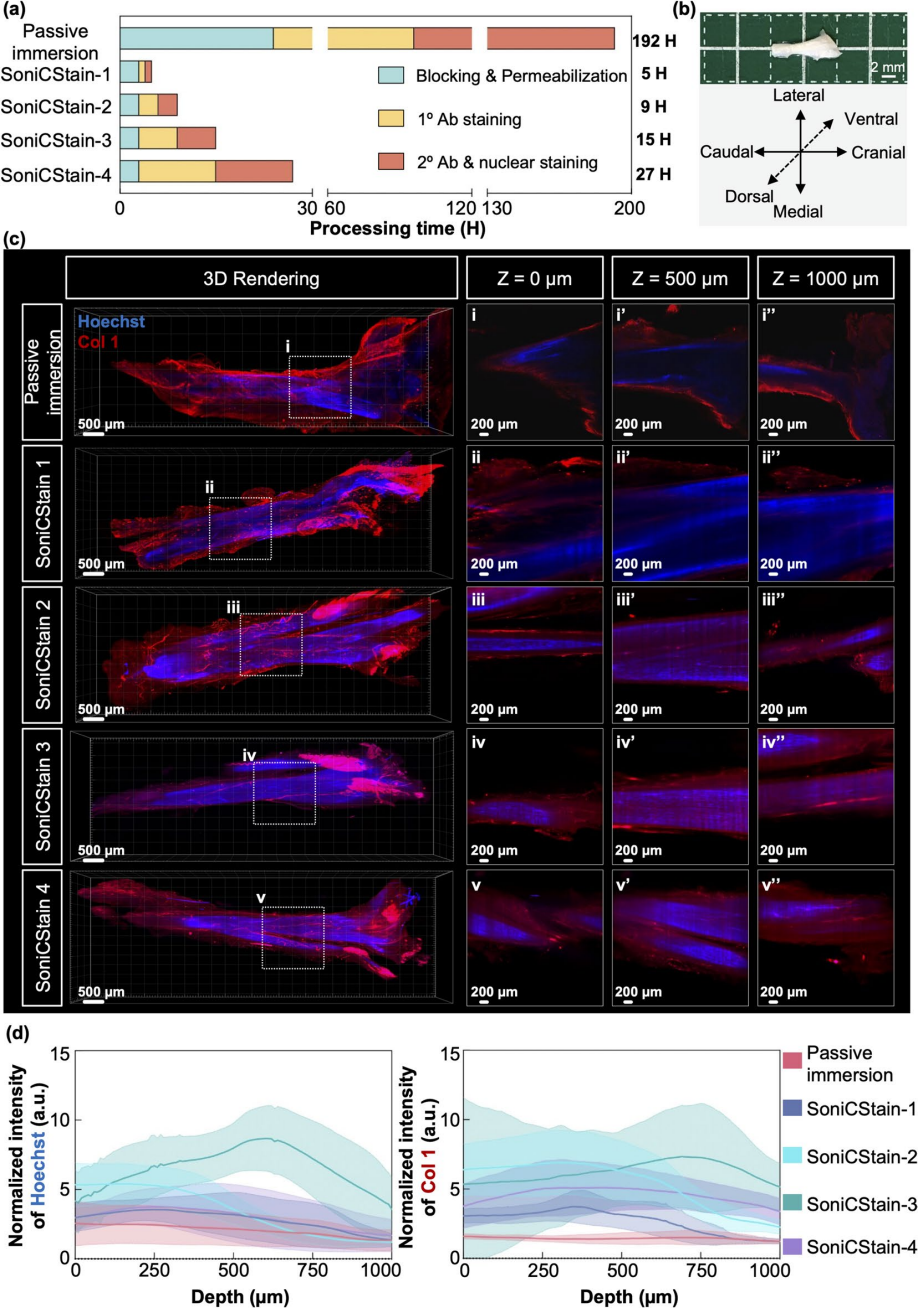
Assessment of imaging depth and signal intensity in rat Achilles tendon using SoniCStain whole-tissue immunofluorescence staining methods. **(a)** Schematic representation of processing durations for passive immersion and SoniCStain methods applied to rat Achilles tendon. **(b)** Macroscopic image depicting the anatomical positioning of the rat Achilles tendon, prepared for microscopic analysis. **(c)** Microscopic images at 4X magnification of whole rat Achilles tendon stained using either passive immersion or SoniCStain methods. The white bounding boxes delineate areas of interest examined at depths of Z = 0 μm, 500 μm, and 1000 μm. Tendons stained via the passive immersion method exhibited fluorescent signals predominantly at the periphery, while the SoniCStain-2, -3, and -4 yielded uniform staining across the tendon samples (blue: Hoechst; red: collagen type I). **(d)** Quantitative analysis of Hoechst and collagen type I (Col I) fluorescent intensities revealed that tendons stained using the SoniCStain method demonstrated significantly higher fluorescent intensity across depths from Z = 0 μm to Z = 1000 μm compared to those stained using passive immersion. (mean ± s.d.; *n* = 3 for each group).

In this study, the SoniCStain protocol significantly reduced the processing time from 192 h in the passive immersion method to a minimum of 5 h for the rat Achilles tendon samples (Fig. 7a). The anatomical positioning of the rat Achilles tendon for microscopic imaging is shown in Fig. 7b. Microscopic analysis revealed that, although the passive immersion method enabled the fluorescent labelling of structures within the 1000 μm thickness of tendon tissue, the normalized signals were predominantly confined to the periphery (Fig. 7c; Supplementary Fig. 5). In contrast, the application of LFU sonication markedly enhanced antibody distribution throughout the tissue (Fig. 7c; Supplementary Fig. 5). The SoniCStain-1 protocol yielded a markedly stronger and more uniform Hoechst signal from the tissue surface (z = 0 μm) to a depth of 1000 μm, compared to the passive immersion group, although a peripheral rimming effect, particularly for the collagen type I (Col 1) signal, was still evident. Furthermore, extending the processing duration enhanced the antibody distribution throughout the tissue. Notably, the SoniCStain-2, 3 and 4 protocol successfully labeled structures up to 1000 μm deep and exhibited a distinguishable Hoechst nuclear signal at a depth of 500 μm from the tissue surface. (Figs. 7c-d; Supplementary Fig. 5). Quantitative analysis of fluorescent intensities demonstrated that the samples processed with SoniCStain methods exhibited higher fluorescent signals for Hoechst and Col 1 from the superficial layer (z = 0 μm) to a depth of 1000 μm, in comparison to samples processed via passive immersion method. For example: SoniCStain-3 achieved the highest fluorescent signals from the superficial layer (z = 0 μm) to depths of 1000 μm, with an average Hoechst signal of 5.4 arbitrary units (a.u.) and an average Col 1 signal of 5.4 a.u. (Fig. 7d). In comparison, the passive immersion method yielded the lowest fluorescent signals with average Hoechst and Col 1 signals of 2.5 a.u. and 1.5 a.u., respectively (Fig. 7d). These results unequivocally indicate that the SoniCStain method facilitates rapid whole-tissue immunofluorescent staining and significantly enhances antibody diffusion within tendon tissues.

## Discussion

**Fig. 8:**
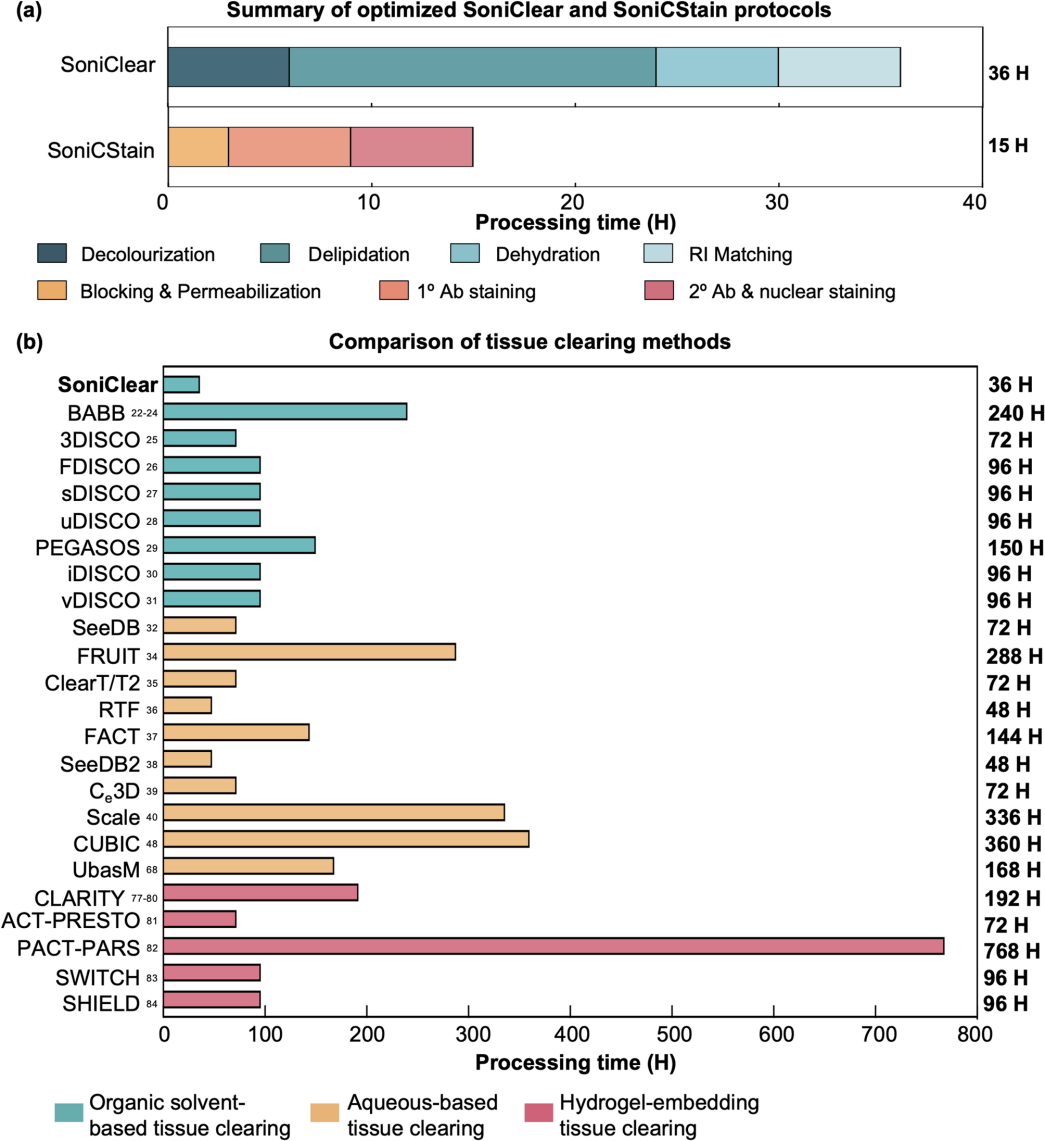
**Summary of the optimized SoniClear and SoniCStain protocols**,** and the comparison of the processing time of different tissue clearing methods.** **(a)** A detailed summary of the optimized protocols for SoniClear and SoniCStain is presented. **(b)** A comparative analysis of the processing times associated with various tissue clearing methods is depicted^22–32,34–40,48,68,77–84^.

In recent years, various optical clearing and whole-tissue staining techniques have been developed. While these methods have successfully achieved high optical transparency and facilitated volumetric imaging, challenges persist, particularly regarding prolonged processing times and Limited diffusion depths of fluorescent probes. In this study, our findings demonstrate the effectiveness of SoniClear in clearing rat Achilles tendons, mouse TA muscles, and spleens for whole tissue imaging. The application of SoniClear resulted in a transparency and light transmittance comparable to that of commercial tissue clearing kits within 36 h while maintaining minimal tissue deformation, which is 4-times faster than the standard processing time of the commercial kit. Furthermore, employing SoniCStain, an enhanced staining performance in dense collagenous tendon tissue was observed, achieving uniform and deep labeling in 15 h, which is 12.8-times faster than the conventional iDISCO method. Collectively, SoniC/S offers a rapid solution for tissue clearing and whole-tissue immunofluorescent staining in soft tissue, dense collagenous tissue as well as heme-rich tissue (Fig. 8a).

Conventional tissue clearing methods necessitate prolonged processing times, often extending from days to several months (Fig. 8b; Supplementary Table 5). To address these time constraints, researchers have developed active tissue clearing techniques employing electric fields^34,36–39^ and perfusion-assisted methodologies^35^. Although these approaches generally result in reduced processing times, the fastest protocol still require a minimum of two days. Additionally, concerns regarding tissue distortion and protein loss during the clearing process raise questions about the reliability of anatomical data obtained through these novel techniques. Therefore, in our study, the effect of LFU sonication on tissue deformation and protein loss was first analyzed. Previous investigations have indicated that prolonged exposure to sonication using high-frequency ultrasound at high intensity (> 1 W/cm^2^), can lead to tissue deformation^53,55,56,64^. Our analyses revealed that LFU sonication at 40 kHz with low intensity (0.370 W/cm^2^) has a negligible impact on the structural integrity of the rat Achilles tendon, mouse TA muscle, and mouse spleen. Although a trend towards increased deformation was observed after 7 days, deformation percentages in the sonication groups remained statistically similar to the control. In contrast to gentle shaking, a method frequently utilized in traditional tissue clearing protocols such as PEGASOS, the deformation percentages in the sonication group were consistently lower (Fig. 2a). After integrating LFU sonication into our SoniClear method, a degree of tissue deformation comparable to the control group without tissue clearing could be observed. In contrast, previous studies associated both passive and active tissue clearing methods with tissue distortion. A tissue expansion of 22.0% was documented in 300-µm-thick brain slices treated with the active CLARITY method. Tissue expansion for passive clearing methods was recorded as 15.9% for CUBIC, 24.5% for ScaleS, and a shrinkage of 13.5% for SeeDB^65^. Subsequently, our study investigated protein loss after LFU sonification and demonstrated a minimal protein loss with values comparable to the control across all tissue types. Specifically, after 7 days of LFU sonication, the protein loss measured ranged from 0.75 ± 0.23 µg/mg (0.075 ± 0.023% loss) in tendon to 8.59 ± 0.37 µg/mg (0.859 ± 0.037% loss) in spleen. In contrast, other active clearing techniques, exemplified by CLARITY, which utilizes an electric field, resulted in approximately 8% protein loss from whole mouse brain tissues during the clearing process^37,66^. Thus, our technique resulted in lower protein loss compared to other active clearing methods, more closely resembling conventional immersion-based techniques. These techniques, such as the FDISCO method, lost approximately 10 µg of protein per mg of tissue from half-coronal 1 mm brain slices following a 7-day incubation in tissue clearing buffer at 37 °C with gentle shaking^67^. These findings suggest that LFU sonication can preserve tissue architecture and may serve as a beneficial enhancement to conventional tissue clearing methods.

This study demonstrates that SoniClear significantly reduces processing time and enhances tissue transparency and light transmittance across various biological samples. Previous research has extensively characterized various tissue clearing approaches. Among them, PACT tissue clearing has been reported to achieve maximum tissue transparency with a value of 10.8 mean Line-pairs per mm for mouse muscle with processing time from 10 to 12 days^51^. Our application of SoniClear yielded substantially higher transparency level for mouse TA muscle as quantified using the same methodology. A tissue transparency of 23.48 mean line-pairs per mm was achieved in SoniClear-1, which required a processing duration of 6 h. Upon extending the processing time, tissue transparency improved to 35.05-line pairs per millimeter (Supplementary Table 6). Subsequently, literature has shown that the inherent vascularization and pigmentation of the spleen, primarily attributed to hemoglobin and other pigments, contribute to significant light absorption, presenting a challenge for conventional clearing strategies^28,68,69^. A recent study has reported that among the tissue clearing methods, PACT achieved maximum tissue transparency values of 16.6 mean line-pairs per mm for mouse spleen^51^. In contrast, our SoniClear method, particularly SoniClear-3, achieves the highest optical clarity within just 36 h, maintaining a transparency of 30.26 mean line-pairs per mm. Furthermore, clearing dense collagenous tissues has proven challenging due to the tightly packed collagen fibers. Studies indicate that such dense structures restrict water diffusion—crucial for the effective penetration of the clearing agents—and the viscoelastic properties of the collagen cross-linking critically affect diffusion dynamics^14,70,71^. Recent data has demonstrated that dense collagenous tissues, including tendons and ligaments, remain opaque even after undergoing optical clearing through clearing methodologies, such as Visikol, ClearT2, and FocusClear^72,73^. In our study, the SoniClear method demonstrated a remarkable enhancement in tissue transparency, allowing for the visualization of underlying grid lines. The quantification of the tissue transparency showed in rat Achilles tendon achieved by SoniClear-4, surpassing even that of the TA muscle. Collectively, our findings indicate that the SoniClear not only enhances tissue transparency and light transmittance but does so in a significantly shorter processing time.

Despite the advancements in tissue transparency, challenges for whole tissue imaging regarding the Limited diffusion depth of fluorescent probes remain. Our SoniCStain method enhanced the penetration of fluorescent antibodies into dense collagenous tendon tissue, achieving depths of up to 1000 μm from the tissue surface. The SoniCStain-3 variant, with a total processing time of 15 h, yielded the highest normalized fluorescent intensity among the examined SoniCStain methods. This contrasts with the conventional iDISCO staining, which required 3–7 days to stain a mouse brain at a depth of 0.5 mm, and exhibited limited fluorescent penetration with peripheral rimming effects^29^. Moreover, literature demonstrates that several alternative approaches have been employed to enhance deep immunostaining capabilities. One such method (eFLASH) employed gradual alterations in detergent micelle concentrations to facilitate antibody diffusion and enhanced the antigen binding by buffer acidification over time^74^. Although it offers high uniformity and consistent staining across brain tissues, it necessitates specialized equipment and customized setups for each specimen. Additionally, optimal results take 24 to 48 h, and its application in dense collagenous tissues has not yet been reported. Another method, ELAST, transforms tissues into elastic gels that can be stretched into thin films, thereby reducing diffusion distances of fluoroprobes^75^. However, this method requires substantial modifications to tissue properties and necessitates a total processing time of 3 to 7 days, with only moderate uniformity of staining^75^. Compared to these techniques, SoniCstain achieved effective staining of collagen rich tissue in a shorter time frame without requiring specialized equipment or tissue modification. Other successful whole-tissue staining of dense collagenous tissues, particularly tendons, are limited. One study by Marr et al. conducted whole-tissue immunofluorescent staining with passive immersion on rat tendon, but fluorescent signals predominantly localized to the peripheral regions^76^. In contrast, our study utilizing SoniCStain-3 demonstrated uniform staining in rat Achilles tendons, with no rimming effects (Fig. 7). Therefore, this method represents a significant advancement in deep tissue immunostaining, presenting a viable solution to overcome the limitations of existing techniques.

This study introduces SoniC/S, a novel technique that enhances the efficacy of tissue clearing and whole-tissue immunofluorescent staining. While we have tested this method on three different tissue types, future studies can explore a broader range of tissues to enhance the generalizability of our findings. This is crucial, as we observed that tissue processing times required are specific to each tissue type. Moreover, our study utilized only two fluorescent probes for labeling. However, multiplex imaging with multiple probes is commonly employed to visualize various targets within a single tissue sample. Future research can encompass a wider array of fluorescent probes to optimize this protocol. Lastly, the parameters for LFU sonication in this study were predetermined according to the manufacturer’s specifications. The effects of varying LFU parameters, including frequency and power settings, have not been thoroughly explored in this study, although these variations may reduce processing duration and enhance the efficacy of tissue clearing and staining.

In conclusion, the SoniC/S method represents a rapid approach to tissue clearing and deep immunostaining. Our findings indicate that this methodology achieved significant tissue transparency, enhanced light transmittance, and increased fluorescence intensity, while reducing tissue deformation and protein loss in adult mouse TA muscle, mouse spleen, and rat Achilles tendon. Future investigations are warranted to explore the compatibility of SoniC/S with multiplexed immunostaining techniques, other tissue clearing and immunostaining protocols, and molecular labeling approaches.

## Supplementary Information

Below is the link to the electronic supplementary material.

Supplementary Material 1

## Data Availability

The datasets generated during and/or analysed during the current study are available from the corresponding author on reasonable request.
