# Supplementary material for: Rapid sonication-assisted whole tissue clearing and immunostaining
Source: Sci Rep. 2025 Oct 8;15:35101. doi: 10.1038/s41598-025-18928-5 (PMC12508232; doi:10.1038/s41598-025-18928-5)
Supplement: Supplementary file 1 — Supplementary Material 1 [file 41598_2025_18928_MOESM1_ESM.pdf]

Supplemental information

Rapid Sonication-Assisted Whole Tissue Clearing and Immunostaining

Hoi Pan Harry Cheung,[1,2,3] Marianne Lauwers,[3] Zhengao Wang,[4] Jianfeng Wang,[5] Chengyun Ning,[6] Dai Fei Elmer Ker,[7] Dan Michelle Wang[1,2,3,8,*]

[1] School of Biomedical Sciences, Faculty of Medicine, The Chinese University of Hong Kong, Sha Tin, New Territories, Hong Kong SAR, China.

[2] Institute for Tissue Engineering and Regenerative Medicine, The Chinese University of Hong Kong, Sha Tin, New Territories, Hong Kong SAR, China.

[3] Center for Neuromusculoskeletal Restorative Medicine, Hong Kong Science Park, Shatin, New Territories, Hong Kong SAR, China.

[4] Research Center of Biomass 3D Printing Materials, College of Materials and Energy, South China Agricultural University, Guangzhou, 510642, China.

[5] College of Materials Science, Hunan University, Changsha, 410082, China.

[6] School of Materials Science and Engineering, National Engineering Research Center for Tissue Restoration and Reconstruction, South China University of Technology, Guangzhou, 510641, China.

[7] Department of Biomedical Engineering, Hong Kong Polytechnic University, Hung Hom, Kowloon, Hong Kong SAR, China.

[8] Department of Orthopaedics and Traumatology, Faculty of Medicine, The Chinese University of Hong Kong, Sha Tin, New Territories, Hong Kong SAR, China.

*Correspondence: wangmd@cuhk.edu.hk

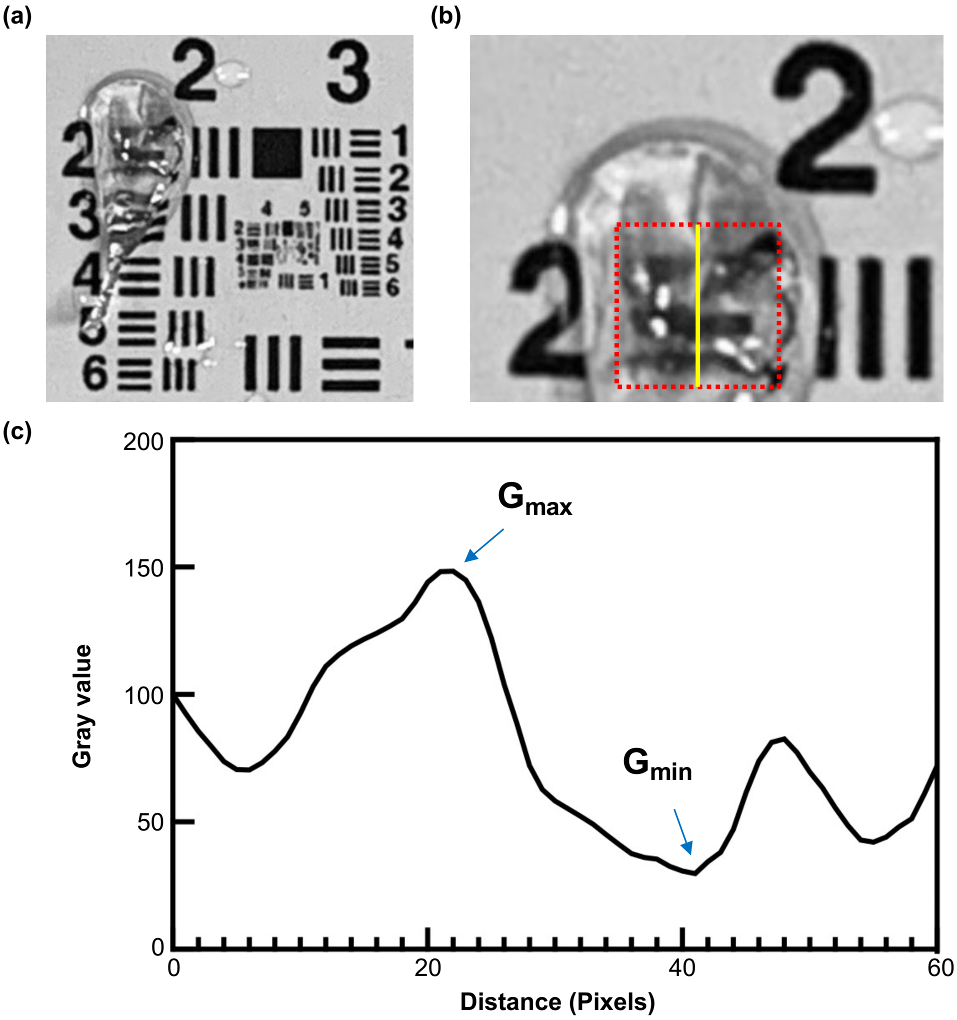

Supplementary Figure 1. The schematic diagram of transparency calculation based on the 1951 USAF target
(a) Transmitted light image of whole mouse TA muscle cleared with PEGASOS. **(b)** Gray value was calculated from yellow vertical lines across regions indicated with red bounding box by Fiji. **(c)** The ratio of gray value in valley (G_{min}) and peak (G_{max}) was calculated.

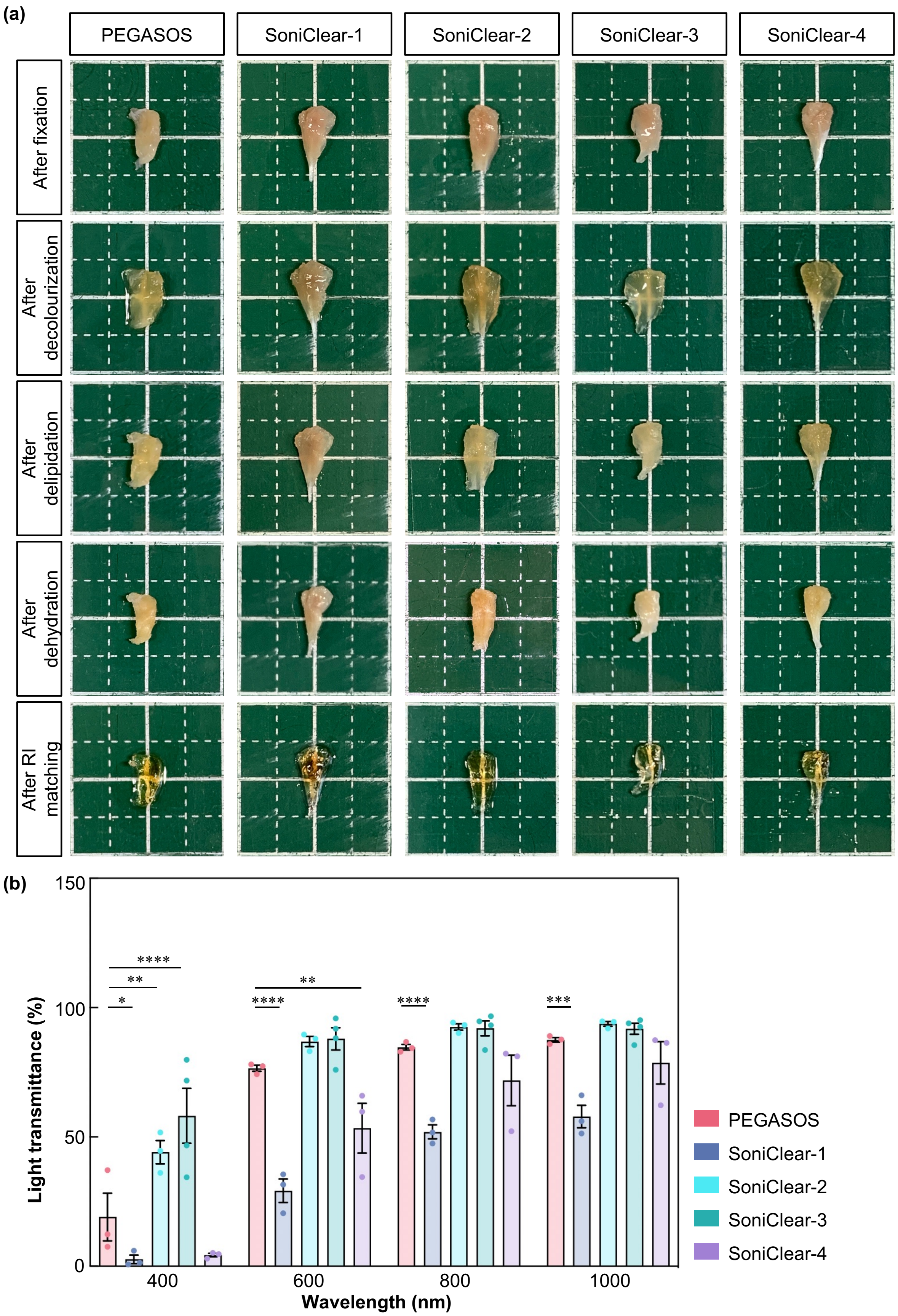

Supplementary Figure 2. Changes in tissue transparency and light transmittance in mouse TA muscle during PEGASOS and SoniClear clearing methods

(a) Representative gross images illustrating the alterations in tissue transparency of mouse TA muscle throughout the PEGASOS and the SoniClear methods. Grid dimensions are 5 mm × 5 mm (n = 3 for each group). **(b)** Quantitative analysis of light transmittance in mouse TA muscle samples (1 mm × 1 mm × 1 mm) processed via PEGASOS and SoniClear treatments, measured at wavelengths of 400 nm, 600 nm, 800 nm, and 1000 nm (mean ± s.d.; n = 3-4 for each group). *, $p < 0.05$; **, $p < 0.01$; ***, $p < 0.001$; ****, $p < 0.0001$.

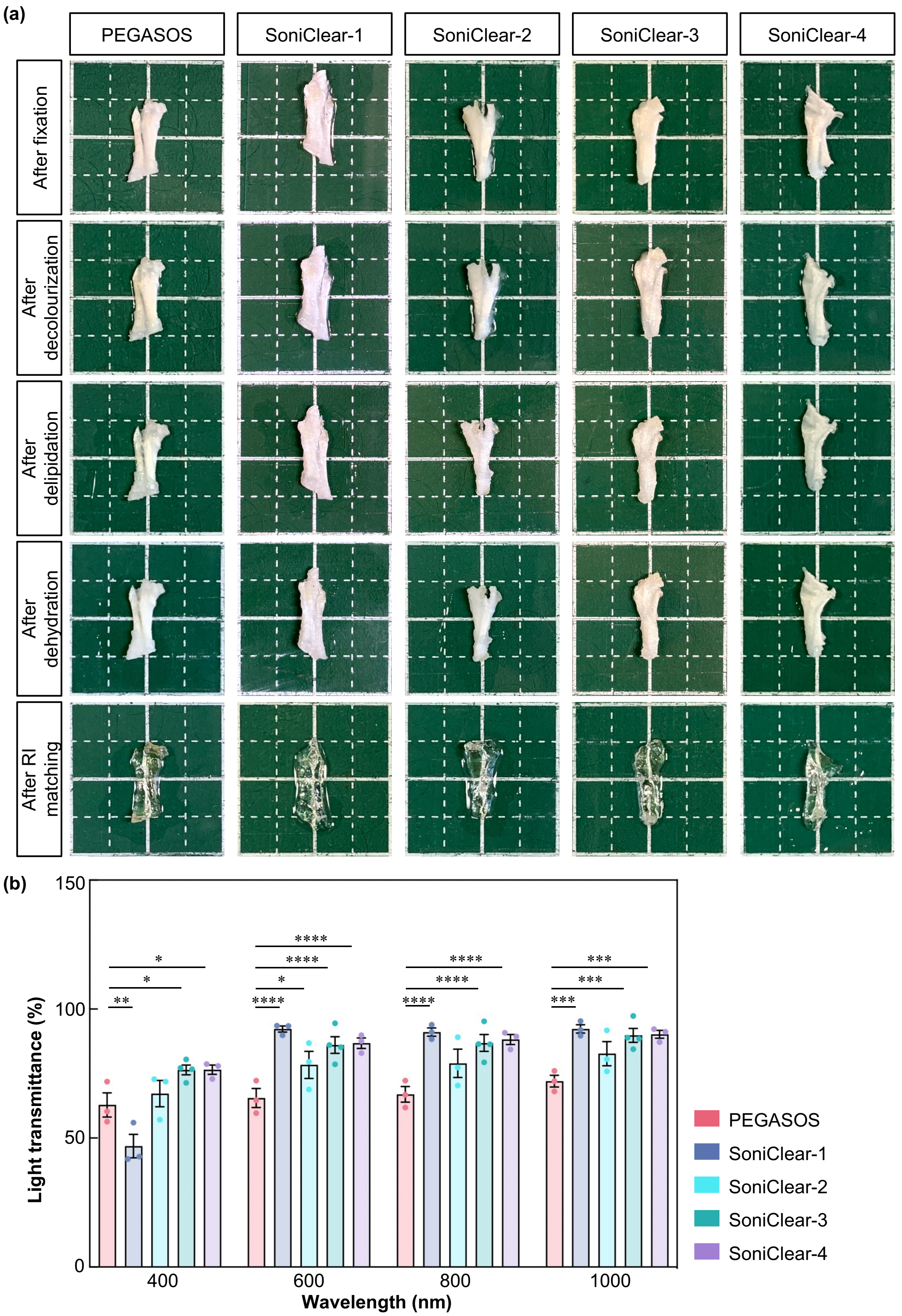

Supplementary Figure 3. Changes in tissue transparency and light transmittance in rat Achilles tendon during PEGASOS and SoniClear clearing methods.

(a) Representative gross images illustrating the alterations in tissue transparency of rat Achilles tendon throughout the PEGASOS and the SoniClear methods. Grid dimensions are 5 mm × 5 mm (n = 3 for each group). **(b)** Quantitative analysis of light transmittance in rat Achilles tendon samples (1 mm × 1 mm × 1 mm) processed via PEGASOS and SoniClear treatments, measured at wavelengths of 400 nm, 600 nm, 800 nm, and 1000 nm (mean ± s.d.; n = 3-4 for each group). *, $p < 0.05$; **, $p < 0.01$; ***, $p < 0.001$; ****, $p < 0.0001$.

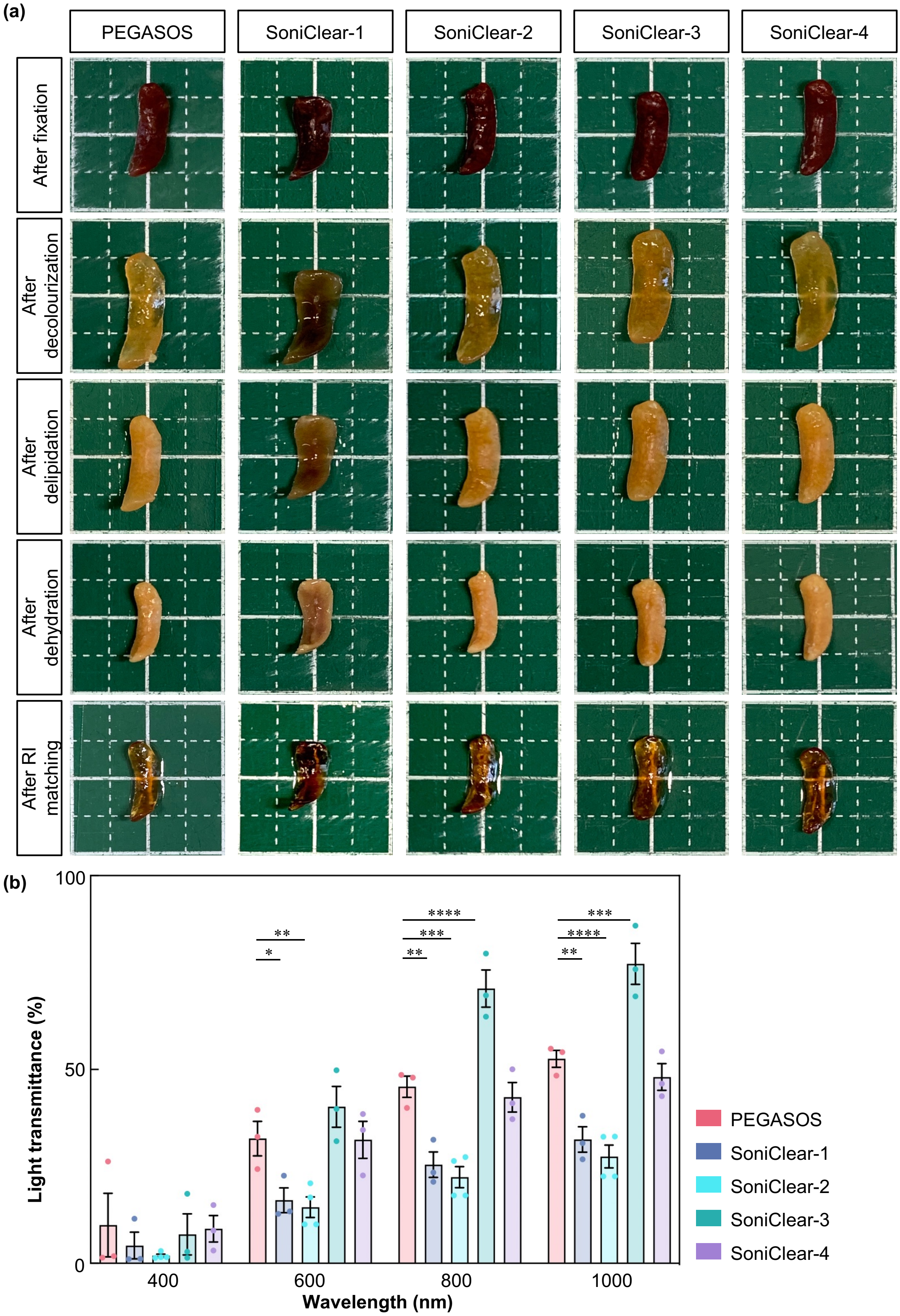

Supplementary Figure 4. Changes in tissue transparency and light transmittance in mouse spleen during PEGASOS and SoniClear clearing methods

(a) Representative gross images illustrating the alterations in tissue transparency of mouse spleen throughout the PEGASOS and the SoniClear. Grid dimensions are 5 mm × 5 mm (n = 3 for each group). **(b)** Quantitative analysis of light transmittance in mouse spleen samples (1 mm × 1 mm × 1 mm) processed via PEGASOS and SoniClear treatments, measured at wavelengths of 400 nm, 600 nm, 800 nm, and 1000 nm (mean ± s.d.; n = 3-4 for each group). *, $p < 0.05$; **, $p < 0.01$; ***, $p < 0.001$; ****, $p < 0.0001$.

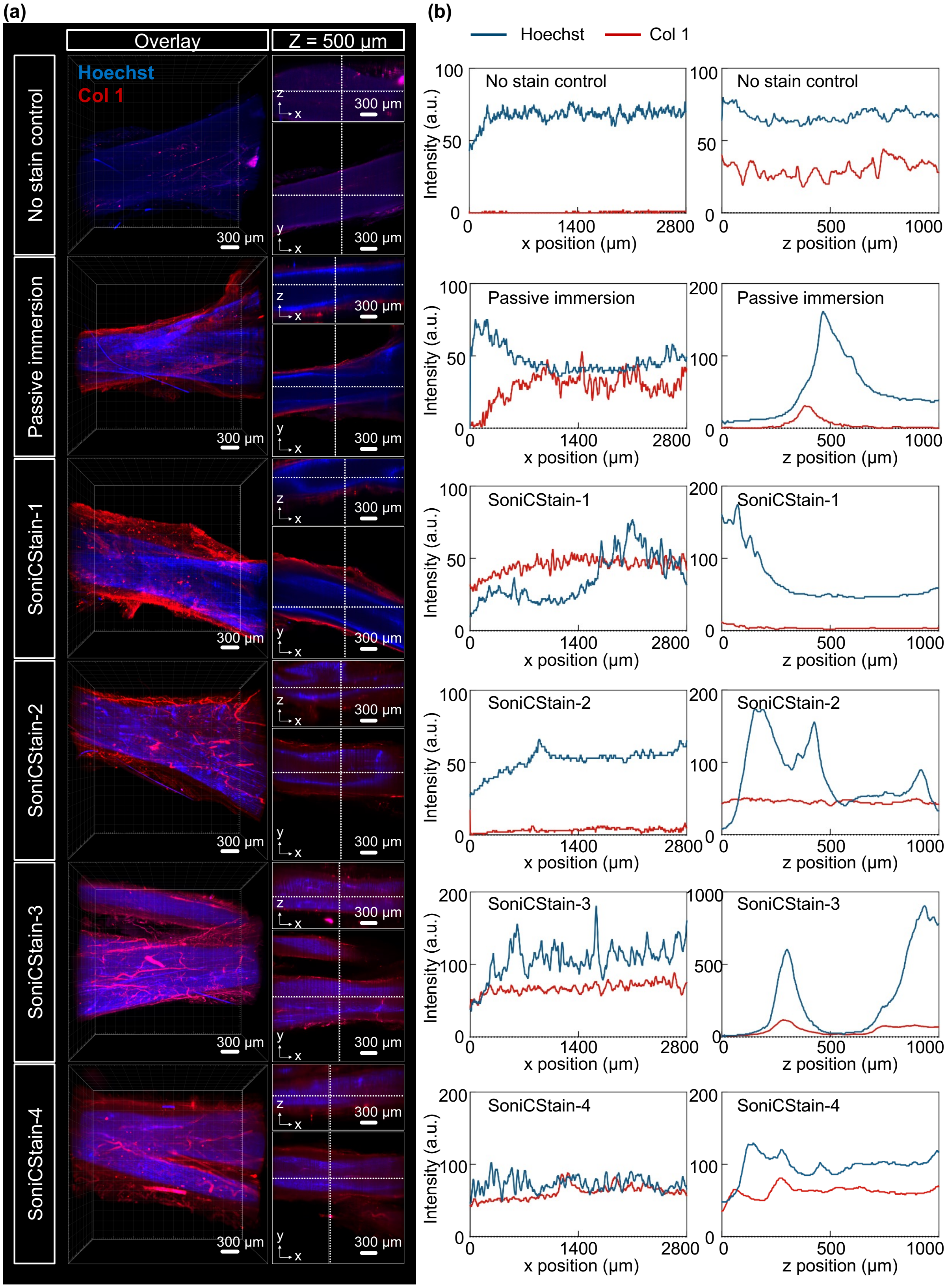

Supplementary Figure 5. Evaluation of staining performance of passive immersion and SoniCStain methods in rat Achille's tendon

(a) Microscopic images at 4X magnification showing the Achilles tendon: no stained, stained using passive immersion, and stained using the SoniCStain method (blue: Hoechst; red: collagen type I). **(b)** Intensity profiles corresponding to the white dashed lines in the 2D images presented in panel (a) (blue: Hoechst; red: collagen type I).

Supplementary Table 1. PEGASOS immersion method time schedule for whole tissue optical clearing

<table>
<tr><th colspan="2"></th><th>Leads Tissue Clearing Solution Kit-PEGASOS (Solution number)</th><th>Processing time (hour)</th><th>Temperature (ºC)</th><th>Gentle shaking (rpm)</th></tr>
<tr><td>Decolorization</td><td>25% Quadrol + Ammonium solution</td><td>2</td><td>48</td><td rowspan="6">37</td><td rowspan="6">220</td></tr>
<tr><td rowspan="3">Delipidation</td><td>30% tert-butanol</td><td>3</td><td>6</td></tr>
<tr><td>50% tert-butanol</td><td>4</td><td>12</td></tr>
<tr><td>70% tert-butanol</td><td>5</td><td>24</td></tr>
<tr><td>Dehydration</td><td>tB-PEG</td><td>6</td><td>24</td></tr>
<tr><td>RI Matching</td><td>BB-PEG</td><td>7</td><td>48</td></tr>
</table>

Supplementary Table 2. Reagents used in SoniClear protocols

Process	Reagent	Leads Tissue Clearing Solution Kit-PEGASOS (Solution number)
Pre-incubation for decolorization (hours)	25% Quadrol + Ammonium solution	2
Decolorization	25% Quadrol + Ammonium solution	2
Delipidation	30% tert-butanol	3
	50% tert-butanol	4
	70% tert-butanol	5
Dehydration	tB-PEG	6
RI Matching	BB-PEG	7

Supplementary Table 3. SoniClear protocols processing time for whole tissue optical clearing

Tissue type	Protocol	Temperature (ºC)	Pre-incubation for decolorization (hours)	Decolorization (hours)	Delipidation (hours)			Dehydration (hours)	RI matching (hours)
					30 %	50 %	70 %		
Rat Achilles tendon and mouse TA muscle	SoniClear-1	37	0	1	1	1	1	1	1
	SoniClear-2		0	3	3	3	3	3	3
	SoniClear-3		0	6	6	6	6	6	6
	SoniClear-4		0	12	12	12	12	12	12
Mouse spleen	SoniClear-1	37	3	1	1	1	1	1	1
	SoniClear-2		3	3	3	3	3	3	3
	SoniClear-3		3	6	6	6	6	6	6
	SoniClear-4		3	12	12	12	12	12	12

Supplementary Table 4. SoniCStain protocols time schedule for whole tissue immunofluorescent staining

Tissue type	Protocol	Temperature (ºC)	Processing time of blocking and permeabilization (hours)	Processing time of 1º Antibody staining (hours)	Processing time of 2º Antibody and nuclear staining (hours)
Rat Achilles tendon, mouse tibialis anterior muscle and spleen	SoniCStain-1	4	3	1	1
	SoniCStain-2		3	3	3
	SoniCStain-3		3	6	6
	SoniCStain-4		3	12	12

Supplementary Table 5. Comparison of the processing time of the existing tissue clearing techniques

Category of tissue clearing	Method	Active / Passive Clearing	Tissue	Processing time
Organic solvent-based tissue clearing	SoniClear *	Active clearing with low-frequency ultrasound applied	Whole rat Achilles' tendon, mouse tibialis anterior muscle and spleen	36 hours
	BABB [1-3]	Passive clearing with immersion	Whole young mouse brain, mouse skin, rat heart and lung, mouse brain, tumor and kidney	10 days
	3DISCO [4]	Passive clearing with immersion	Whole mouse mammary gland, lymph node, spinal cord, lung, spleen, brain stem, brain and pancreas	3 days
	FDISCO [5]	Passive clearing with gentle shaking	Whole adult mouse brain	3-4 days
	sDISCO [6]	Passive clearing with gentle shaking	Whole adult mouse brain	4 days
	uDISCO [7]	Passive clearing with gentle shaking and rotation	Whole mouse body without skin	3-4 days
	PEGASOS [8]	Passive clearing with immersion	Whole mouse body with skin	14 days
	iDISCO [9]	Passive clearing with immersion	Mouse embryo, hole-brain, spinal cord, brain, lung and pancreas	4 days
	vDISCO [10]	Passive clearing with immersion	Whole-body	4 days
Aqueous-based tissue clearing	SeeDB [11]	Passive clearing with thermal energy	Mouse hemi-brain	3 days
	FRUIT [12]	Passive clearing with gentle rotation	Whole adult rabbit Brain	12 days
	ClearT/T2 [13]	Passive clearing with immersion	Mouse brain and embryo	3 days
	RTF [14]	Passive clearing with immersion	Adult mouse brain blocks	2 days
	FACT [15]	Passive clearing with gentle rotation	Whole adult mouse brain	3-9 days
	SeeDB2 [16]	Passive clearing with immersion	Mouse hemi-brain	2 days
	C$_e$3D [17]	Passive clearing with immersion	Whole lymph node	3 days
	Scale [18]	Passive clearing with immersion	Adult mouse brain section, and mouse embryo	14 days
	CUBIC [19]	Passive clearing with gentle shaking	Whole adult mouse	15-19 days
	UbasM [20]	Passive clearing with gentle shaking	Mouse hemi-brain	7 days
Hydrogel-embedding tissue clearing	CLARITY [21-24]	Active clearing with electric field applied	Mouse brain, human brain (postmortem), mouse brain, pancreas, kidney, lung, intestine and liver, spinal cord	8-22 days
	ACT-PRESTO [25]	Active clearing with electric field applied	Mouse and rabbit brain, mouse thymus, intestine, testis, lung, spleen, liver and kidney	2-3 days
	PACT-PARS [26]	Active clearing with perfusion-assisted	Whole mouse body, Mouse brain, kidney, lung, heart, intestine and tumor	32 days
	SWITCH [27]	Passive clearing with thermal energy	Adult rat and young marmoset brains	4-14 days
	SHIELD [28]	Active clearing with stochastic electrotransport	Mouse brain human block	4-6 days

*: Tissue clearing method developed in this study

Supplementary Table 6. Quantitative assessment of transparency for whole organs with PEGASOS and SoniClear methods

Mean Line-pairs per mm						
	1xPBS	PEGASOS	SoniClear 1	SoniClear 2	SoniClear 3	SoniClear 4
Mouse tibialis anterior muscle	0	15.13	23.48	28.61	31.23	35.05
Rat Achilles tendon	0	32.00	31.13	32.10	38.10	44.33
Mouse spleen	0	19.43	16.98	28.64	30.26	28.64

The USAF resolution target was used and mean numbers (line-pairs per mm) were calculated to represent levels of transparency (n = 3 for each group).

Supplemental Reference

1 Dodt, H. U. *et al.* Ultramicroscopy: three-dimensional visualization of neuronal networks in the whole mouse brain. *Nat Methods* **4**, 331-336, doi:10.1038/nmeth1036 (2007).

2 Foster, D. S. *et al.* A Clearing Technique to Enhance Endogenous Fluorophores in Skin and Soft Tissue. *Sci Rep* **9**, 15791, doi:10.1038/s41598-019-50359-x (2019).

3 Oldham, M., Sakhalkar, H., Oliver, T., Allan Johnson, G. & Dewhirst, M. Optical clearing of unsectioned specimens for three-dimensional imaging via optical transmission and emission tomography. *J Biomed Opt* **13**, 021113, doi:10.1117/1.2907968 (2008).

4 Erturk, A. *et al.* Three-dimensional imaging of solvent-cleared organs using 3DISCO. *Nat Protoc* **7**, 1983-1995, doi:10.1038/nprot.2012.119 (2012).

5 Qi, Y. *et al.* FDISCO: Advanced solvent-based clearing method for imaging whole organs. *Sci Adv* **5**, eaau8355, doi:10.1126/sciadv.aau8355 (2019).

6 Hahn, C. *et al.* High-resolution imaging of fluorescent whole mouse brains using stabilised organic media (sDISCO). *J Biophotonics* **12**, e201800368, doi:10.1002/jbio.201800368 (2019).

7 Pan, C. *et al.* Shrinkage-mediated imaging of entire organs and organisms using uDISCO. *Nat Methods* **13**, 859-867, doi:10.1038/nmeth.3964 (2016).

8 Jing, D. *et al.* Tissue clearing of both hard and soft tissue organs with the PEGASOS method. *Cell Res* **28**, 803-818, doi:10.1038/s41422-018-0049-z (2018).

9 Renier, N. *et al.* iDISCO: a simple, rapid method to immunolabel large tissue samples for volume imaging. *Cell* **159**, 896-910, doi:10.1016/j.cell.2014.10.010 (2014).

10 Cai, R. *et al.* Panoptic imaging of transparent mice reveals whole-body neuronal projections and skull-meninges connections. *Nat Neurosci* **22**, 317-327, doi:10.1038/s41593-018-0301-3 (2019).

11 Ke, M. T., Fujimoto, S. & Imai, T. SeeDB: a simple and morphology-preserving optical clearing agent for neuronal circuit reconstruction. *Nat Neurosci* **16**, 1154-1161, doi:10.1038/nn.3447 (2013).

12 Hou, B. *et al.* Scalable and DiI-compatible optical clearance of the mammalian brain. *Front Neuroanat* **9**, 19, doi:10.3389/fnana.2015.00019 (2015).

13 Kuwajima, T. *et al.* ClearT: a detergent- and solvent-free clearing method for neuronal and non-neuronal tissue. *Development* **140**, 1364-1368, doi:10.1242/dev.091844 (2013).

14 Yu, T. *et al.* RTF: a rapid and versatile tissue optical clearing method. *Sci Rep* **8**, 1964, doi:10.1038/s41598-018-20306-3 (2018).

15 Xu, N. *et al.* Fast free-of-acrylamide clearing tissue (FACT)-an optimized new protocol for rapid, high-resolution imaging of three-dimensional brain tissue. *Sci Rep* **7**, 9895, doi:10.1038/s41598-017-10204-5 (2017).

16 Ke, M. T. *et al.* Super-Resolution Mapping of Neuronal Circuitry With an Index-Optimized Clearing Agent. *Cell Rep* **14**, 2718-2732, doi:10.1016/j.celrep.2016.02.057 (2016).

17 Li, W., Germain, R. N. & Gerner, M. Y. Multiplex, quantitative cellular analysis in large tissue volumes with clearing-enhanced 3D microscopy (C(e)3D). *Proc Natl Acad Sci U S A* **114**, E7321-E7330, doi:10.1073/pnas.1708981114 (2017).

18 Hama, H. *et al.* Scale: a chemical approach for fluorescence imaging and reconstruction of transparent mouse brain. *Nat Neurosci* **14**, 1481-1488, doi:10.1038/nn.2928 (2011).

19 Susaki, E. A. *et al.* Whole-brain imaging with single-cell resolution using chemical cocktails and computational analysis. *Cell* **157**, 726-739, doi:10.1016/j.cell.2014.03.042 (2014).

20 Chen, L. *et al.* UbasM: An effective balanced optical clearing method for intact biomedical imaging. *Sci Rep* **7**, 12218, doi:10.1038/s41598-017-12484-3 (2017).

21 Chung, K. *et al.* Structural and molecular interrogation of intact biological systems. *Nature* **497**, 332-337, doi:10.1038/nature12107 (2013).

22 Tomer, R., Ye, L., Hsueh, B. & Deisseroth, K. Advanced CLARITY for rapid and high-resolution imaging of intact tissues. *Nat Protoc* **9**, 1682-1697, doi:10.1038/nprot.2014.123 (2014).

23 Lee, H., Park, J. H., Seo, I., Park, S. H. & Kim, S. Improved application of the electrophoretic tissue clearing technology, CLARITY, to intact solid organs including brain, pancreas, liver, kidney, lung, and intestine. *BMC Dev Biol* **14**, 48, doi:10.1186/s12861-014-0048-3 (2014).

24 Zhang, M. D. *et al.* Neuronal calcium-binding proteins 1/2 localize to dorsal root ganglia and excitatory spinal neurons and are regulated by nerve injury. *Proc Natl Acad Sci U S A* **111**, E1149-1158, doi:10.1073/pnas.1402318111 (2014).
25 Lee, E. *et al.* ACT-PRESTO: Rapid and consistent tissue clearing and labeling method for 3-dimensional (3D) imaging. *Sci Rep* **6**, 18631, doi:10.1038/srep18631 (2016).
26 Yang, B. *et al.* Single-cell phenotyping within transparent intact tissue through whole-body clearing. *Cell* **158**, 945-958, doi:10.1016/j.cell.2014.07.017 (2014).
27 Murray, E. *et al.* Simple, Scalable Proteomic Imaging for High-Dimensional Profiling of Intact Systems. *Cell* **163**, 1500-1514, doi:10.1016/j.cell.2015.11.025 (2015).
28 Park, Y. G. *et al.* Protection of tissue physicochemical properties using polyfunctional crosslinkers. *Nat Biotechnol*, doi:10.1038/nbt.4281 (2018).
